# Supplementary material for: Odoribacter splanchnicus rescues aging-related intestinal P-glycoprotein damage via GDP-L-fucose secretion
Source: Nat Commun. 2025 Nov 27;16:10665. doi: 10.1038/s41467-025-65692-1 (PMC12660754; doi:10.1038/s41467-025-65692-1)
Supplement: Supplementary file 1 — Supplementary Information [file 41467_2025_65692_MOESM1_ESM.pdf]

Supplementary Figures, Tables and References

Figure S1 3

Figure S2 5

Figure S3 4

Figure S4 2

Figure S5 6

Figure S6 9

Figure S7 11

Figure S8 13

Figure S9 15

Figure S10 17

Figure S11 19

Figure S12 21

Figure S13 23

Figure S14 25

Figure S15 27

Figure S16 30

Figure S17 33

Figure S18 29

Table S1 34

Table S2 35

Table S3 36

Table S4 38

Table S5 39

Supplementary references 40

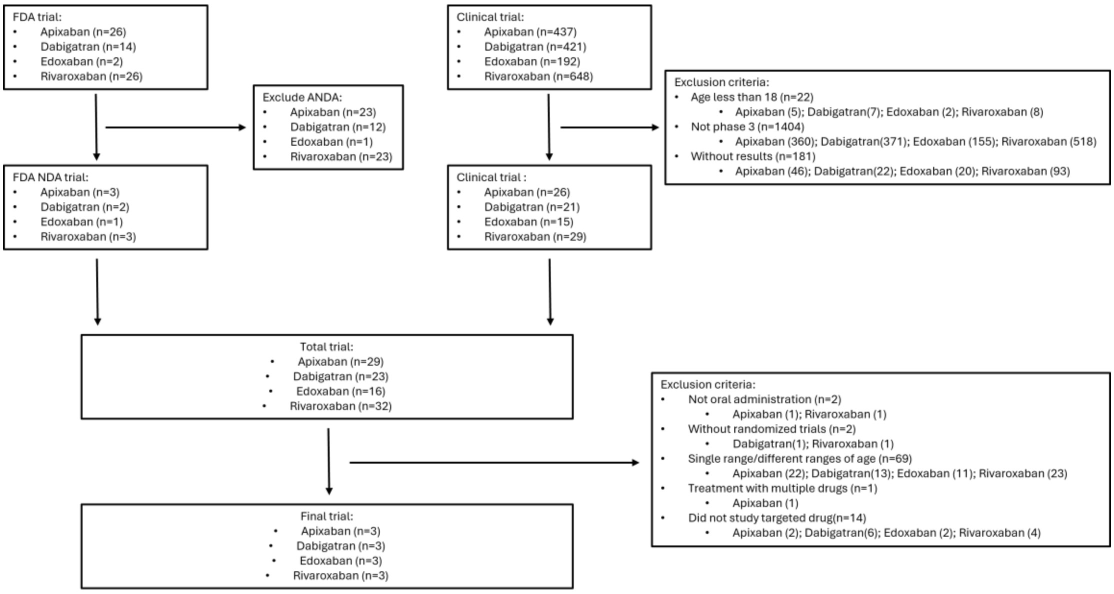

Figure S1

DOAC bleeding risk and age meta-analysis workflow.

Study or Subgroup	age <75 Events	age <75 Total	age≥75 Events	age≥75 Total	Weight	Risk Ratio M-H, Fixed, 95% CI
**1.1.1 Dabigatran**						
NCT00262600(110mg)	138	10855	204	7258	10.6%	0.45 [0.37, 0.56]
NCT00262600(150mg)	153	10855	246	7258	12.8%	0.42 [0.34, 0.51]
NCT02239120(110mg)	57	2147	20	548	1.4%	0.73 [0.44, 1.20]
NDA 22-512(110mg)	138	3666	204	2349	10.8%	0.43 [0.35, 0.53]
NDA 22-512(150mg)	153	3610	246	2466	12.7%	0.42 [0.35, 0.52]
**Subtotal (95% CI)**		**31133**		**19879**	**48.2%**	**0.44 [0.40, 0.49]**
Total events	639		920			
Heterogeneity: Chi² = 4.39, df = 4 (P = 0.36); I² = 9%						
Test for overall effect: Z = 16.14 (P < 0.00001)						
**1.1.2 Edoxaban**						
NCT00986154(60mg)	279	3558	70	560	5.2%	0.63 [0.49, 0.80]
NCT02866175(30mg和60mg)	73	501	55	250	3.2%	0.66 [0.48, 0.91]
NDA206316(30mg)	121	4213	133	2789	6.9%	0.60 [0.47, 0.77]
NDA206316(60mg)	194	4174	224	2838	11.6%	0.59 [0.49, 0.71]
**Subtotal (95% CI)**		**12446**		**6437**	**26.9%**	**0.61 [0.54, 0.68]**
Total events	667		482			
Heterogeneity: Chi² = 0.46, df = 3 (P = 0.93); I² = 0%						
Test for overall effect: Z = 8.26 (P < 0.00001)						
**1.1.3 Rivaroxaban**						
NCT00403767(20mg)	172	3487	223	3496	9.7%	0.77 [0.64, 0.94]
NCT00440193(15mg)	7	253	1	100	0.1%	2.77 [0.34, 22.20]
NCT02313909(15mg)	55	5520	30	1693	2.0%	0.56 [0.36, 0.87]
**Subtotal (95% CI)**		**9260**		**5289**	**11.7%**	**0.75 [0.63, 0.89]**
Total events	234		254			
Heterogeneity: Chi² = 3.23, df = 2 (P = 0.20); I² = 38%						
Test for overall effect: Z = 3.23 (P = 0.001)						
**1.1.4 Apixaban**						
NCT00496769(2.5mg和5mg)	72	1900	64	906	3.8%	0.54 [0.39, 0.74]
NCT00633893(2.5mg)	10	729	4	111	0.3%	0.38 [0.12, 1.19]
NCT00633893(5mg)	13	704	1	109	0.1%	2.01 [0.27, 15.23]
NDA202155(2.5mg和5mg)	176	6252	151	2836	9.0%	0.53 [0.43, 0.65]
**Subtotal (95% CI)**		**9585**		**3962**	**13.1%**	**0.54 [0.45, 0.64]**
Total events	271		220			
Heterogeneity: Chi² = 2.00, df = 3 (P = 0.57); I² = 0%						
Test for overall effect: Z = 6.97 (P < 0.00001)						
**Total (95% CI)**		**62424**		**35567**	**100.0%**	**0.53 [0.50, 0.57]**
Total events	1811		1876			
Heterogeneity: Chi² = 42.87, df = 15 (P = 0.0002); I² = 65%						
Test for overall effect: Z = 19.02 (P < 0.00001)						
Test for subgroup differences: Chi² = 33.52, df = 3 (P < 0.00001), I² = 91.1%						

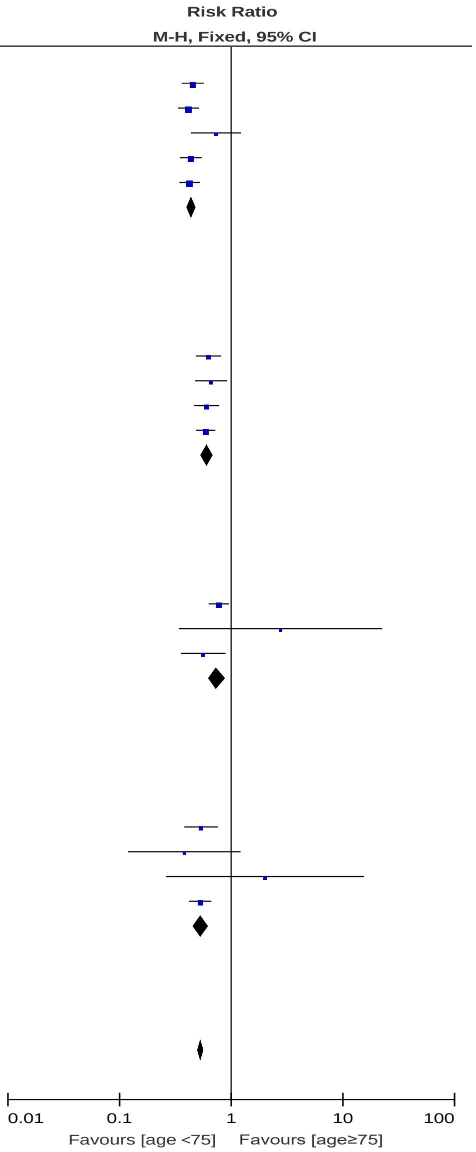

Figure S2

A meta-analysis of age and bleeding risk of novel oral anticoagulants (P-gp substrate drugs).

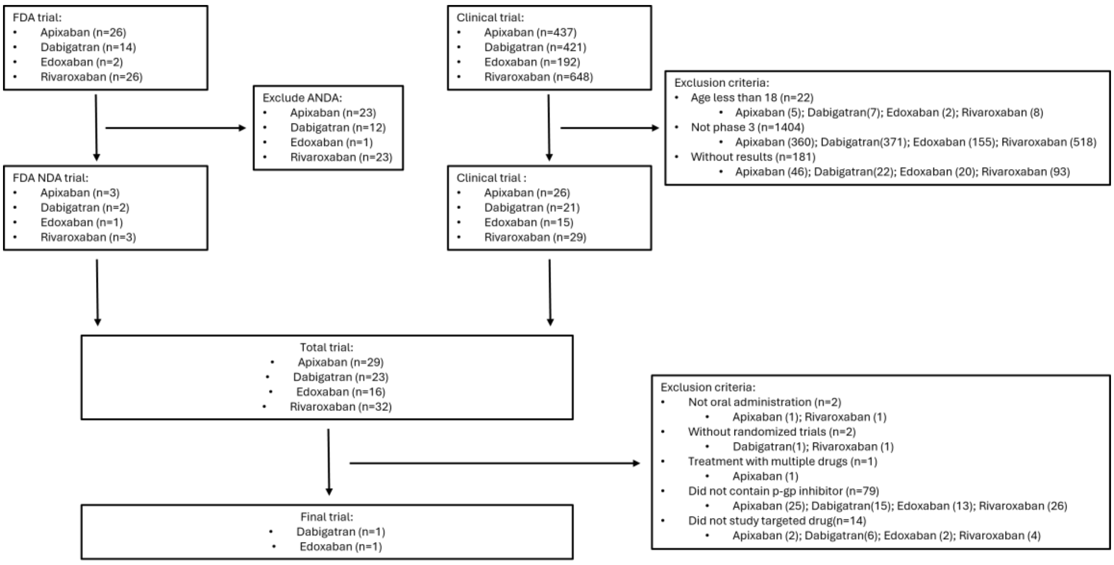

Figure S3

DOAC bleeding risk and P-gp inhibitor meta-analysis workflow.

Study or Subgroup	Experimental Events	Experimental Total	Experimental+inhibitor Events	Experimental+inhibitor Total	Weight	Risk Ratio M-H, Fixed, 95% CI
**1.1.1 Dabigatran**						
NDA 22-512(110mg)-Amiodarone	241	5139	54	844	14.7%	0.73 [0.55, 0.98]
NDA 22-512(110mg)-Diltiazem	240	5310	55	673	15.4%	0.55 [0.42, 0.73]
NDA 22-512(110mg)-Verapamil*	273	5574	22	409	6.5%	0.91 [0.60, 1.39]
NDA 22-512(150mg)-Amiodarone	295	5191	55	868	14.9%	0.90 [0.68, 1.19]
NDA 22-512(150mg)-Diltiazem	302	5384	48	675	13.5%	0.79 [0.59, 1.06]
NDA 22-512(150mg)-Verapamil*	321	5650	29	409	8.5%	0.80 [0.56, 1.16]
**Subtotal (95% CI)**		**32248**		**3878**	**73.5%**	**0.76 [0.67, 0.87]**
Total events	1672		263			
Heterogeneity: Chi² = 7.16, df = 5 (P = 0.21); I² = 30%						
Test for overall effect: Z = 4.21 (P < 0.0001)						
**1.1.2 Edoxaban**						
NDA206316(60mg)-Amiodarone	341	5810	77	1202	20.2%	0.92 [0.72, 1.16]
NDA206316(60mg)-Verapamil*/Quinidine	397	6677	21	335	6.3%	0.95 [0.62, 1.45]
**Subtotal (95% CI)**		**12487**		**1537**	**26.5%**	**0.92 [0.75, 1.14]**
Total events	738		98			
Heterogeneity: Chi² = 0.02, df = 1 (P = 0.89); I² = 0%						
Test for overall effect: Z = 0.74 (P = 0.46)						
**Total (95% CI)**		**44735**		**5415**	**100.0%**	**0.81 [0.72, 0.90]**
Total events	2410		361			
Heterogeneity: Chi² = 9.85, df = 7 (P = 0.20); I² = 29%						
Test for overall effect: Z = 3.93 (P < 0.0001)						
Test for subgroup differences: Chi² = 2.39, df = 1 (P = 0.12), I² = 58.1%						

Risk Ratio
M-H, Fixed, 95% CI

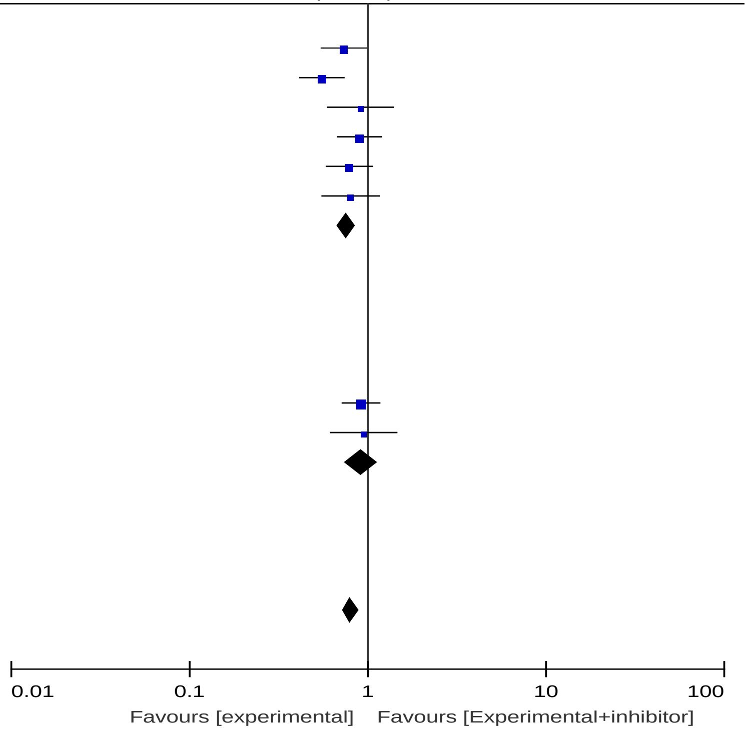

Figure S4

A meta-analysis of P-gp inhibitors and bleeding risk of novel oral anticoagulants (P-gp substrate drugs).

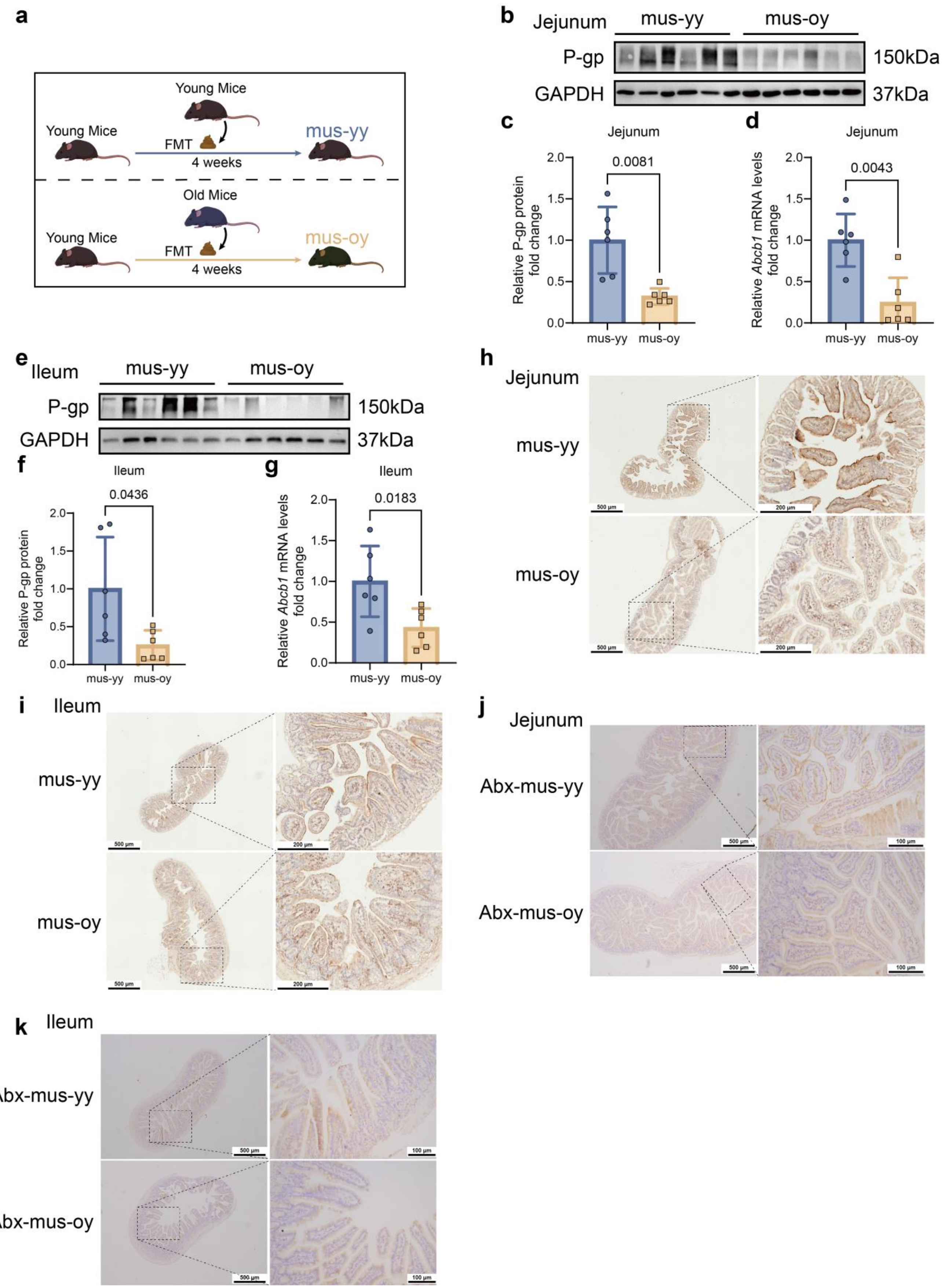
a
Young Mice
Young Mice
FMT
4 weeks
mus-yy
Old Mice
Young Mice
FMT
4 weeks
mus-oy
b
Jejunum
mus-yy
mus-oy
P-gp
150kDa
GAPDH
37kDa
c
Jejunum
0.0081
Relative P-gp protein fold change
mus-yy
mus-oy
d
Jejunum
0.0043
Relative Abcb1 mRNA levels fold change
mus-yy
mus-oy
e
Ileum
mus-yy
mus-oy
P-gp
150kDa
GAPDH
37kDa
f
Ileum
0.0436
Relative P-gp protein fold change
mus-yy
mus-oy
g
Ileum
0.0183
Relative Abcb1 mRNA levels fold change
mus-yy
mus-oy
h
Jejunum
mus-yy
mus-oy
i
Ileum
mus-yy
mus-oy
j
Jejunum
Abx-mus-yy
Abx-mus-oy
k
Ileum
Abx-mus-yy
Abx-mus-oy

Figure S5

Colonization of gut microbiota from elderly mice downregulates intestinal P-gp expression in young mice.

a-i. C57BL/6 WT 12-week-old mice were treated with mus-y-fecal-microbiota-transplantation (mus-yy) or mus-o-fecal-microbiota-transplantation (mus-oy) for 4 weeks (n =6/group).
a. Overview of animal experiments of mus-yy and mus-oy mouse models.
b-c. Expression levels of P-gp in jejunum of mus-yy and mus-oy groups (n =6/group), and quantification.
d. *Abcb1* mRNA expression levels of P-gp in jejunum of mus-yy and mus-oy groups (n =6/group),
e-f. Expression levels of P-gp in ileum of mus-yy and mus-oy groups (n =6/group), and quantification.
g. *Abcb1* mRNA expression levels of P-gp in ileum of mus-yy and mus-oy groups (n =6/group).
h-i. IHC staining of P-gp on jejunum and ileum sections (mus-yy and mus-oy groups, scale bar: 500 and 200 μm).
j-k. IHC staining of P-gp on jejunum and ileum sections (n =6/group) (Abx-mus-yy and Abx-mus-oy groups, scale bar: 500 and 100 μm).

Data are presented as mean ± SD. Statistical analyses were conducted using two-tailed unpaired t-test with Welch's correction (c, f), Mann–Whitney U test (d), and two-tailed unpaired t-test (g). Exact P values are reported in the figures.

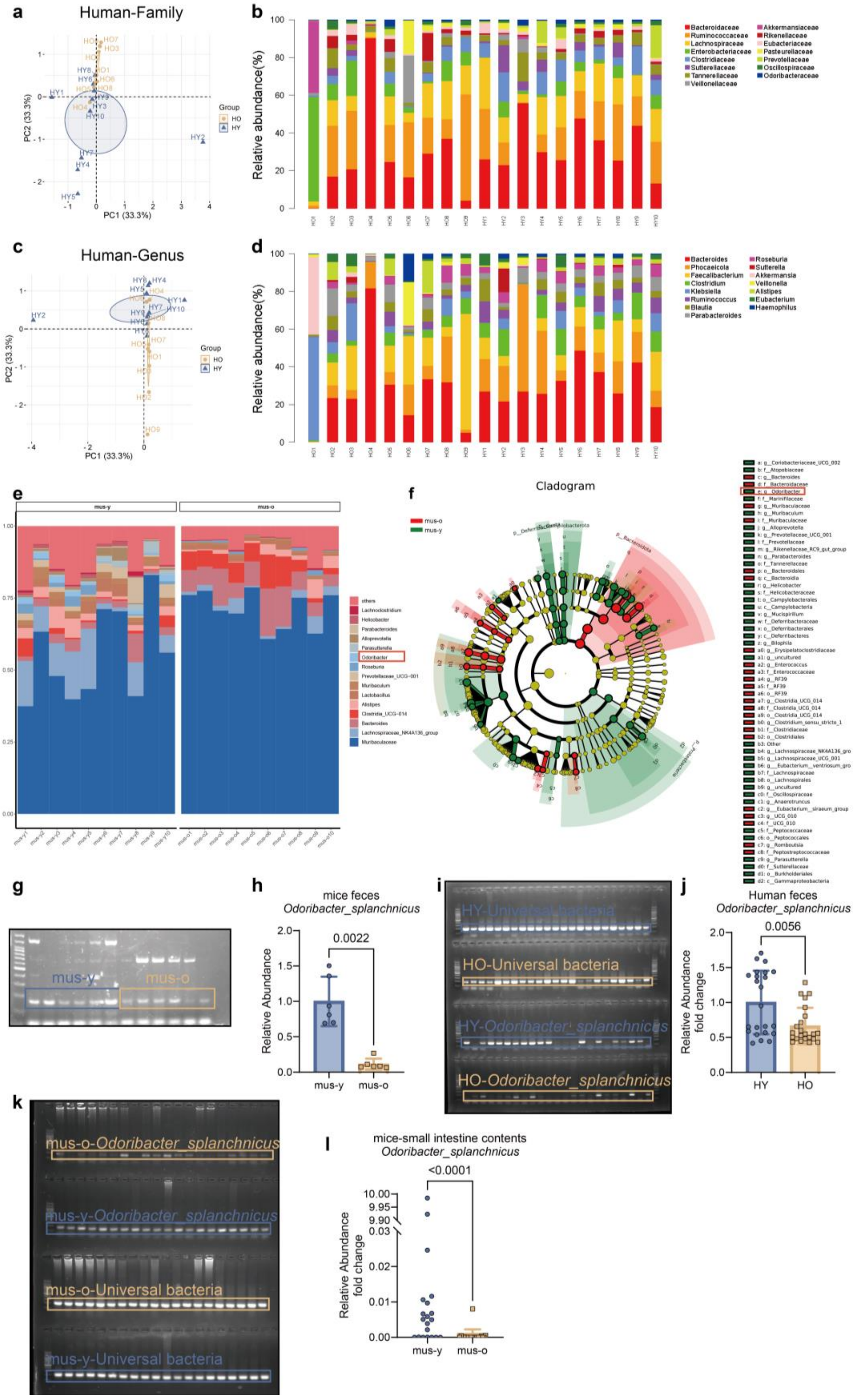
a
Human-Family
PC1 (33.3%)
PC2 (33.3%)
Group
HO
HY
b
Relative abundance(%)
Bacteroidaceae
Ruminococcaceae
Lachnospiraceae
Enterobacteriaceae
Clostridiaceae
Sutterellaceae
Tannerellaceae
Veillonellaceae
Akkermansiaceae
Rikenellaceae
Eubacteriaceae
Pasteurellaceae
Prevotellaceae
Oscillospiraceae
Odoribacteraceae
c
Human-Genus
d
Bacteroides
Phocaeicola
Faecalibacterium
Clostridium
Klebsiella
Ruminococcus
Blautia
Parabacteroides
Roseburia
Sutterella
Akkermansia
Veillonella
Alistipes
Eubacterium
Haemophilus
e
mus-y
mus-o
f
Cladogram
mus-o
mus-y
g
mus-y
mus-o
h
mice feces
Odoribacter_splanchnicus
0.0022
Relative Abundance
mus-y
mus-o
i
HY-Universal bacteria
HO-Universal bacteria
HY-Odoribacter_splanchnicus
HO-Odoribacter_splanchnicus
j
Human feces
Odoribacter_splanchnicus
0.0056
Relative Abundance fold change
HY
HO
k
mus-o-Odoribacter_splanchnicus
mus-y-Odoribacter_splanchnicus
mus-o-Universal bacteria
mus-y-Universal bacteria
l
mice-small intestine contents
Odoribacter_splanchnicus
<0.0001
Relative Abundance fold change
mus-y
mus-o

Figure S6

The gut microbiota diversity in the elderly is markedly distinct from that in younger individuals.

a-d. The Metagenomics gene profiling data for fecal microbiome from HY and HO groups (n =10 and 9/group).
a. PCoA plot of beta-diversity at family level.
b. Relative abundance of significantly altered taxa at the rank of family (including unspecified taxa).
c. PCoA plot of beta-diversity at genus level.
d. Relative abundance of significantly altered taxa at the rank of genus (including unspecified taxa).
e-f. The 16S rRNA gene profiling data for fecal microbiome from mus-y and mus-o groups (n =10/group).
e. Relative abundance of significantly altered taxa at the rank of genus (including unspecified taxa).
f. LEfSe analysis of significantly altered taxa at the rank of genus (including unspecified taxa).
g-h. The abundance and quantitative analysis of *Odoribacter_splanchnicus* (*O. splanchnicus*) in fecal bacteria DNA from mus-y and mus-o groups by PCR (i) and qPCR (j) (n =6/group).
i-j. The abundance and quantitative analysis of *O. splanchnicus* in fecal bacteria DNA from HY and HO groups by PCR (n =23/group).
k-l. The abundance and quantitative analysis of *O. splanchnicus* in small intestine contents bacterial DNA from mus-y and mus-o groups by PCR (k) and qPCR (l) (n =20/group).

Data are presented as mean ± SD. Statistical analyses were conducted using Mann–Whitney U test (h, j, l). Exact P values are reported in the figures.

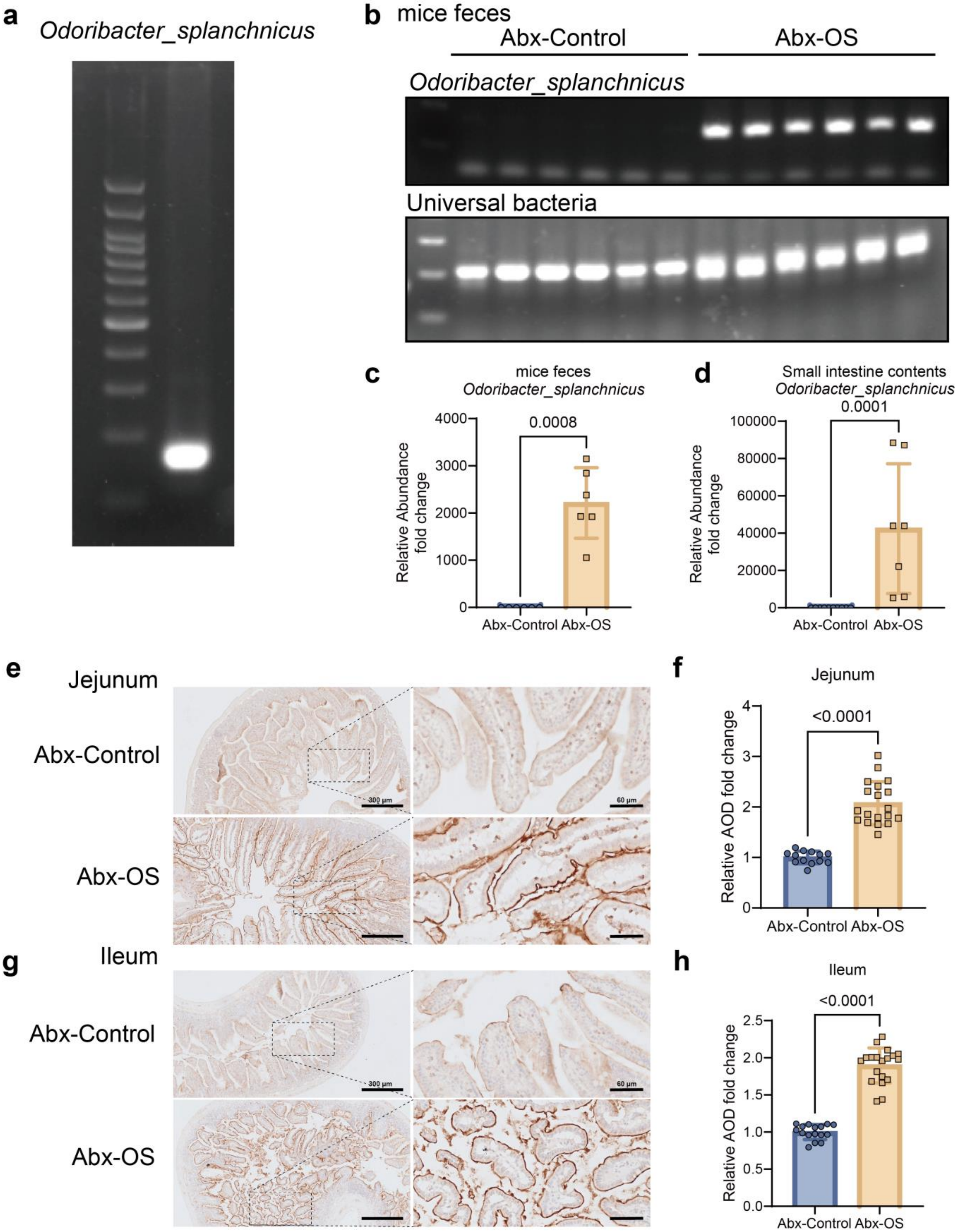
a
Odoribacter_splanchnicus
b
mice feces
Abx-Control
Abx-OS
Odoribacter_splanchnicus
Universal bacteria
c
mice feces
Odoribacter_splanchnicus
0.0008
Relative Abundance fold change
Abx-Control
Abx-OS
d
Small intestine contents
Odoribacter_splanchnicus
0.0001
Relative Abundance fold change
Abx-Control
Abx-OS
e
Jejunum
Abx-Control
Abx-OS
300 μm
60 μm
f
Jejunum
<0.0001
Relative AOD fold change
Abx-Control
Abx-OS
g
Ileum
Abx-Control
Abx-OS
300 μm
60 μm
h
Ileum
<0.0001
Relative AOD fold change
Abx-Control
Abx-OS

Figure S7

Gavage of *O. splanchnicus* to mice treated with Abx can facilitate the colonization into the mice and increase the expression levels of P-gp in the small intestine.

a. Identify *O. splanchnicus* by PCR.
b-d. Sterile PBS control or *O. splanchnicus* were transferred to Abx-treated WT mice. PCR(b) and qPCR(c) with fecal bacteria DNA (n =6/group), and qPCR(d) with small intestine contents bacterial DNA (n =10 and 7/group) to validate the successful colonization.
e-f. IHC staining of P-gp on jejunum sections (Abx-Control and Abx-OS groups, scale bar: 300 and 60 μm), and the analysis of P-gp positive areas (n =13 and 19/group).
g-h. IHC staining of P-gp on ileum sections (Abx-Control and Abx-OS groups, scale bar: 300 and 60 μm), and the analysis of P-gp positive areas (n =16 and 19/group).

Data are presented as mean ± SD. Statistical analyses were conducted using two-tailed unpaired t-test with Welch's correction (c, f, h) and Mann–Whitney U test (d). Exact P values are reported in the figures.

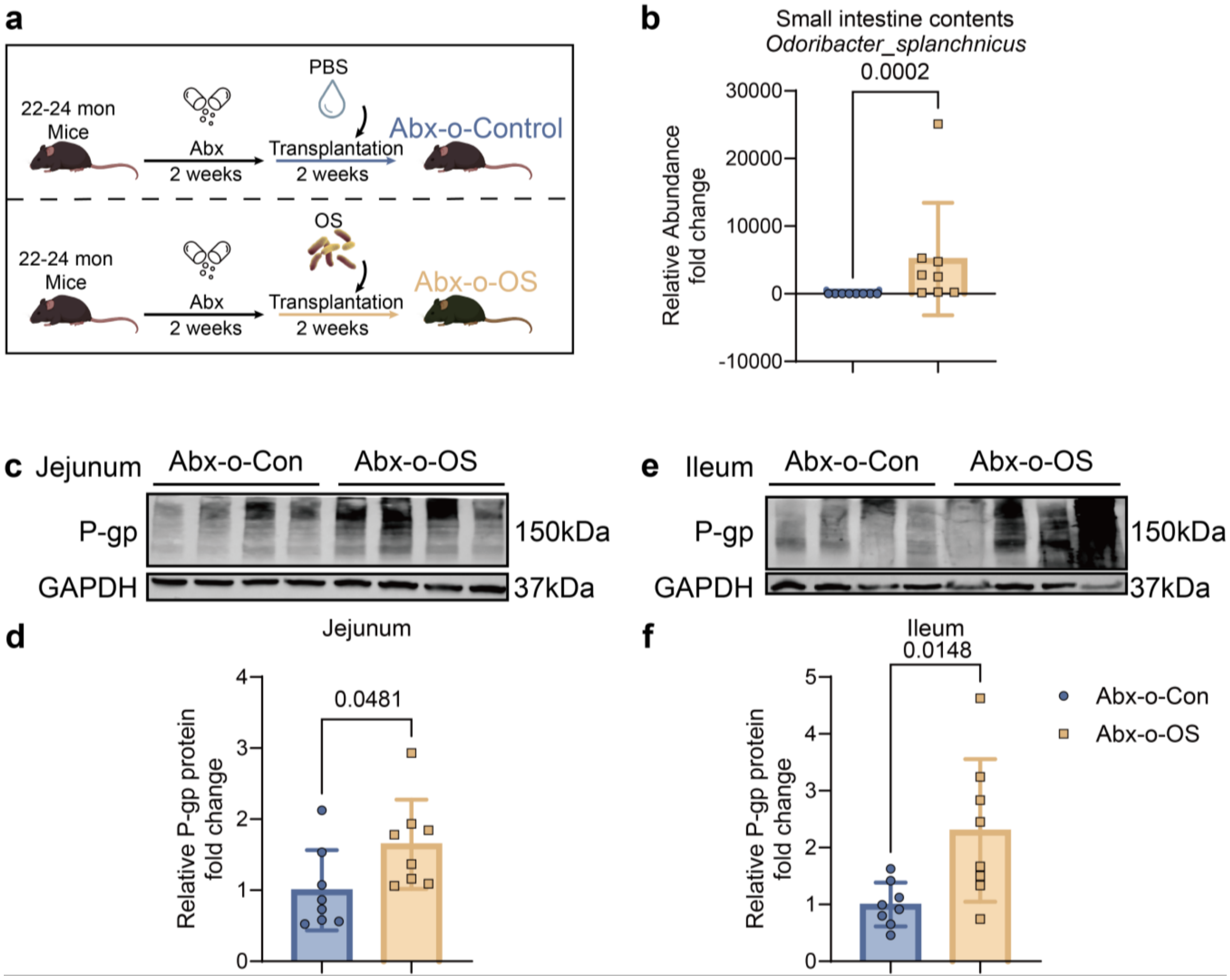
a
22-24 mon Mice
Abx 2 weeks
PBS
Transplantation 2 weeks
Abx-o-Control
OS
Abx-o-OS
b
Small intestine contents
Odoribacter_splanchnicus
0.0002
Relative Abundance fold change
30000
20000
10000
0
-10000
c Jejunum
Abx-o-Con
Abx-o-OS
P-gp
150kDa
GAPDH
37kDa
e Ileum
d
Jejunum
0.0481
Relative P-gp protein fold change
f
Ileum
0.0148
Abx-o-Con
Abx-o-OS

Figure S8

Gavage of *O. splanchnicus* to aged mice treated with Abx can elevate the expression levels of P-gp in their small intestines.

a-f. C57BL/6 WT 22- to 24-month-old mice were treated with Abx for 2 weeks and then with PBS control (Abx-o-Con) or *O. splanchnicus* (Abx-o-OS) for 2 weeks (n =8/group).
a. Overview of animal experiments of Abx-o-Con and Abx-o-OS mouse models.
b. qPCR with small intestine contents bacterial DNA to validate the successful colonization.
c-d. Expression levels of P-gp in jejunum of Abx-o-Con and Abx-o-OS groups and quantification.
e-f. Expression levels of P-gp in ileum of Abx-o-Con and Abx-o-OS groups and quantification.

Data are presented as mean ± SD. Statistical analyses were conducted using Mann–Whitney U test (b, f) and two-tailed unpaired t-test (d). Exact P values are reported in the figures.

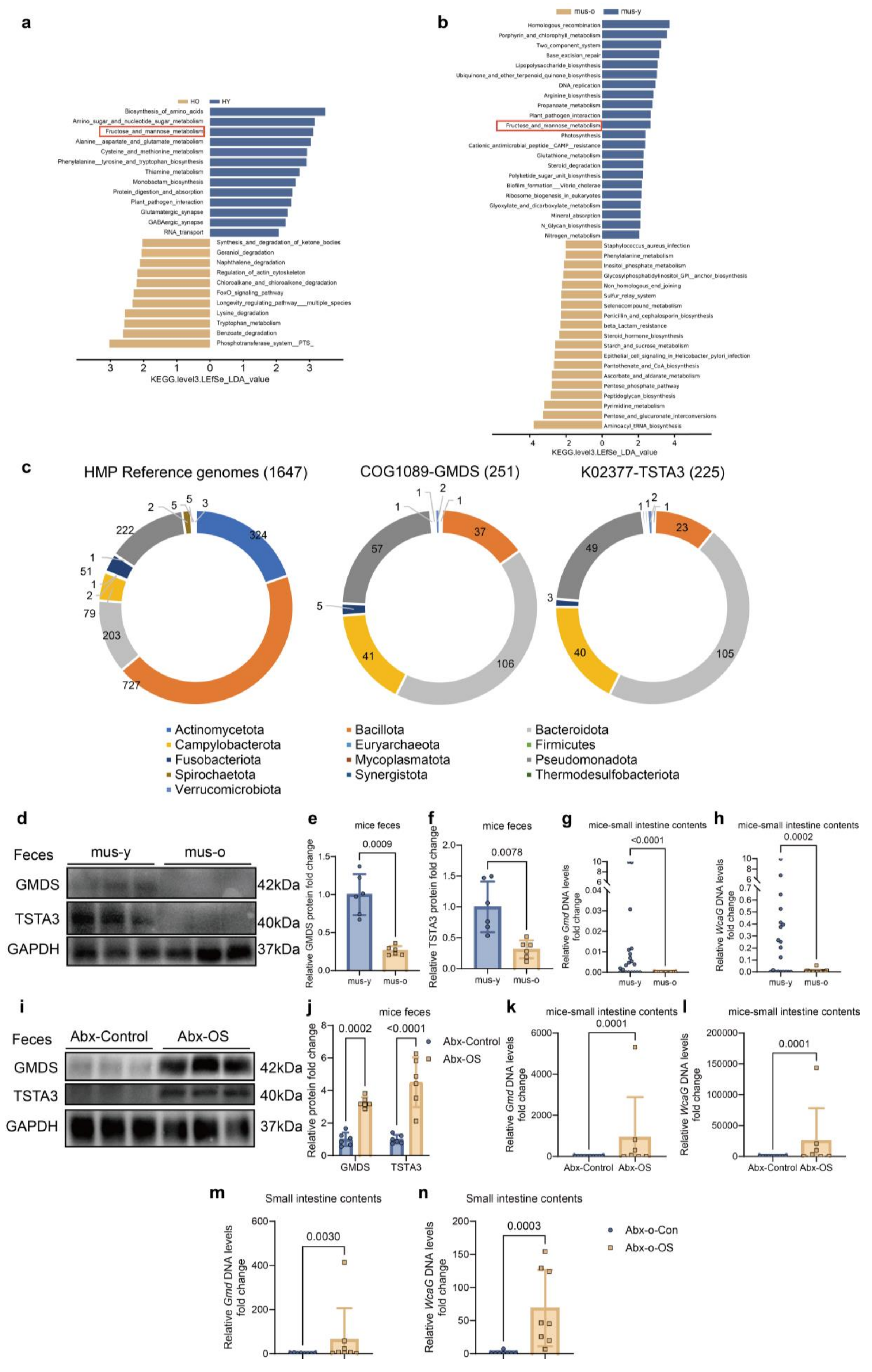

a
HO
HY
Biosynthesis_of_amino_acids
Amino_sugar_and_nucleotide_sugar_metabolism
Fructose_and_mannose_metabolism
Alanine__aspartate_and_glutamate_metabolism
Cysteine_and_methionine_metabolism
Phenylalanine__tyrosine_and_tryptophan_biosynthesis
Thiamine_metabolism
Monobactam_biosynthesis
Protein_digestion_and_absorption
Plant_pathogen_interaction
Glutamatergic_synapse
GABAergic_synapse
RNA_transport
Synthesis_and_degradation_of_ketone_bodies
Geraniol_degradation
Naphthalene_degradation
Regulation_of_actin_cytoskeleton
Chloroalkane_and_chloroalkene_degradation
FoxO_signaling_pathway
Longevity_regulating_pathway__multiple_species
Lysine_degradation
Tryptophan_metabolism
Benzoate_degradation
Phosphotransferase_system__PTS_
KEGG.level3.LEfSe_LDA_value
b
mus-o
mus-y
Homologous_recombination
Porphyrin_and_chlorophyll_metabolism
Two_component_system
Base_excision_repair
Lipopolysaccharide_biosynthesis
Ubiquinone_and_other_terpenoid_quinone_biosynthesis
DNA_replication
Arginine_biosynthesis
Propanoate_metabolism
Plant_pathogen_interaction
Fructose_and_mannose_metabolism
Photosynthesis
Cationic_antimicrobial_peptide__CAMP__resistance
Glutathione_metabolism
Steroid_degradation
Polyketide_sugar_unit_biosynthesis
Biofilm_formation__Vibrio_cholerae
Ribosome_biogenesis_in_eukaryotes
Glyoxylate_and_dicarboxylate_metabolism
Mineral_absorption
N_Glycan_biosynthesis
Nitrogen_metabolism
Staphylococcus_aureus_infection
Phenylalanine_metabolism
Inositol_phosphate_metabolism
Glycosylphosphatidylinositol_GPI__anchor_biosynthesis
Non_homologous_end_joining
Sulfur_relay_system
Selenocompound_metabolism
Penicillin_and_cephalosporin_biosynthesis
beta_Lactam_resistance
Steroid_hormone_biosynthesis
Starch_and_sucrose_metabolism
Epithelial_cell_signaling_in_Helicobacter_pylori_infection
Pantothenate_and_CoA_biosynthesis
Ascorbate_and_aldarate_metabolism
Pentose_phosphate_pathway
Peptidoglycan_biosynthesis
Pyrimidine_metabolism
Pentose_and_glucuronate_interconversions
Aminoacyl_tRNA_biosynthesis
KEGG.level3.LEfSe_LDA_value
c
HMP Reference genomes (1647)
COG1089-GMDS (251)
K02377-TSTA3 (225)
Actinomycetota
Bacillota
Bacteroidota
Campylobacterota
Euryarchaeota
Firmicutes
Fusobacteriota
Mycoplasmatota
Pseudomonadota
Spirochaetota
Synergistota
Thermodesulfobacteriota
Verrucomicrobiota
d
Feces
mus-y
mus-o
GMDS
TSTA3
GAPDH
42kDa
40kDa
37kDa
e
mice feces
Relative GMDS protein fold change
0.0009
f
mice feces
Relative TSTA3 protein fold change
0.0078
g
mice-small intestine contents
Relative Gmd DNA levels fold change
<0.0001
h
mice-small intestine contents
Relative WcaG DNA levels fold change
0.0002
i
Feces
Abx-Control
Abx-OS
j
mice feces
Relative protein fold change
0.0002
<0.0001
GMDS
TSTA3
k
mice-small intestine contents
Relative Gmd DNA levels fold change
0.0001
l
mice-small intestine contents
Relative WcaG DNA levels fold change
0.0001
m
Small intestine contents
Relative Gmd DNA levels fold change
0.0030
n
Small intestine contents
Relative WcaG DNA levels fold change
0.0003
Abx-o-Con
Abx-o-OS

Figure S9

Young mice's feces and small intestine contents exhibit high expression of GMDS and TSTA3.

a. The KEGG level3 LEfSe analysis of metagenomic genes for fecal microbiome from HY and HO groups (n = 10 and 9/group).
b. The KEGG level3 LEfSe analysis of 16S rRNA gene profiling data for fecal microbiome from mus-y and mus-o groups (n = 10/group).
c. Distribution of GMDS and TSTA3 in Human Microbiome Project (HMP) reference genomes. The pie charts show the total number of microbial genomes harboring the corresponding subject (GMDS and TSTA3) classified according to the phyla. The leftmost chart shows the total number of microbial genomes included in the analyses for each phylum. Analyses were performed using the COG and KEGG functions; COG1089 (for GMDS) and K02377 (for TSTA3), while K01711 (for GMDS) was discarded due to low data accuracy.
d-f. Expression levels of GMDS and TSTA3 in feces of mus-y and mus-o groups (n=6/group), and quantification.
g-h. Detection of *Gmd* and *WcaG* levels in bacterial DNA from small intestinal contents of mus-y and mus-o groups by qPCR (n=20/group).
i-j. Expression levels of GMDS and TSTA3 in feces of Abx-Control and Abx-OS groups (n=6/group), and quantification.
k-l. Detection of *Gmd* and *WcaG* levels in bacterial DNA from small intestinal contents of Abx-Control and Abx-OS groups by qPCR (n=10 and 7/group).
m-n. Detection of *Gmd* and *WcaG* levels in bacterial DNA from small intestinal contents of Abx-o-Con and Abx-o-OS groups by qPCR (n=8/group).

Data are presented as mean ± SD. Statistical analyses were conducted using two-tailed unpaired t-test with Welch's correction (e, f), Mann-Whitney U test (g, h, k, l, m, n), and two-way ANOVA (j). Exact P values are reported in the figures.

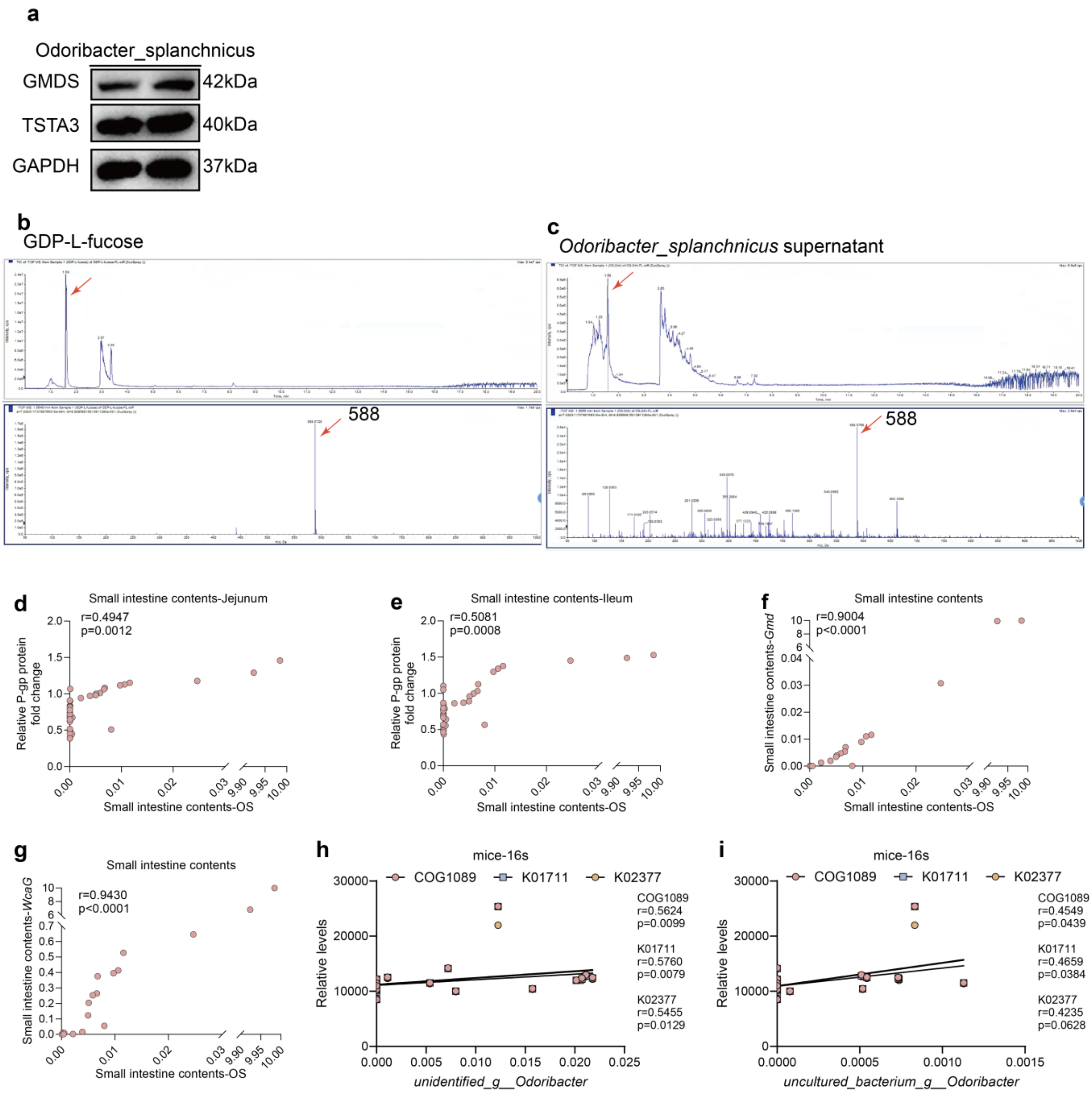
a
Odoribacter_splanchnicus
GMDS
42kDa
TSTA3
40kDa
GAPDH
37kDa
b
GDP-L-fucose
588
c
Odoribacter_splanchnicus supernatant
588
d
Small intestine contents-Jejunum
r=0.4947
p=0.0012
Relative P-gp protein fold change
Small intestine contents-OS
e
Small intestine contents-Ileum
r=0.5081
p=0.0008
Relative P-gp protein fold change
Small intestine contents-OS
f
Small intestine contents
r=0.9004
p<0.0001
Small intestine contents-Gmd
Small intestine contents-OS
g
Small intestine contents
r=0.9430
p<0.0001
Small intestine contents-WcaG
Small intestine contents-OS
h
mice-16s
COG1089
K01711
K02377
COG1089
r=0.5624
p=0.0099
K01711
r=0.5760
p=0.0079
K02377
r=0.5455
p=0.0129
Relative levels
unidentified_g__Odoribacter
i
mice-16s
COG1089
K01711
K02377
COG1089
r=0.4549
p=0.0439
K01711
r=0.4659
p=0.0384
K02377
r=0.4235
p=0.0628
Relative levels
uncultured_bacterium_g__Odoribacter

Figure S10

***O. splanchnicus* expresses GMDS and TSTA3, metabolizes GDP-L-fucose, and its abundance positively correlates with small intestinal P-gp expression.**

a. Expression levels of GMDS and TSTA3 in *O. splanchnicus*.
b-c. Identify and detect GDP-L-fucose standard and GDP-L-fucose in *O. splanchnicus* supernatant by targeted liquid chromatography-mass spectrometry (LC/MS).
d. Correlation between *O. splanchnicus* abundance in small intestinal contents and jejunal P-gp expression of mus-y and mus-o groups (n=20/group).
e. Correlation between *O. splanchnicus* abundance in small intestinal contents and ileum P-gp expression of mus-y and mus-o groups (n=20/group).
f. Correlation between *O. splanchnicus* abundance in small intestinal contents and *Gmd* DNA levels of mus-y and mus-o groups (n=20/group).
g. Correlation between *O. splanchnicus* abundance in small intestinal contents and *WcaG* DNA levels of mus-y and mus-o groups (n=20/group).
h. Correlation between *unidentified_g__Odoribacter* abundance in fecal 16S analysis and levels of COG1089, K01711, K02377 of mus-y and mus-o groups (n=10/group).
i. Correlation between *uncultured_bacterium_g__Odoribacter* abundance in fecal 16S analysis and levels of COG1089, K01711, K02377 of mus-y and mus-o groups (n=10/group).

Correlation analysis: Normality assessed by Shapiro-Wilk test; Pearson correlation used for normally distributed data, Spearman rank correlation for non-normal data.

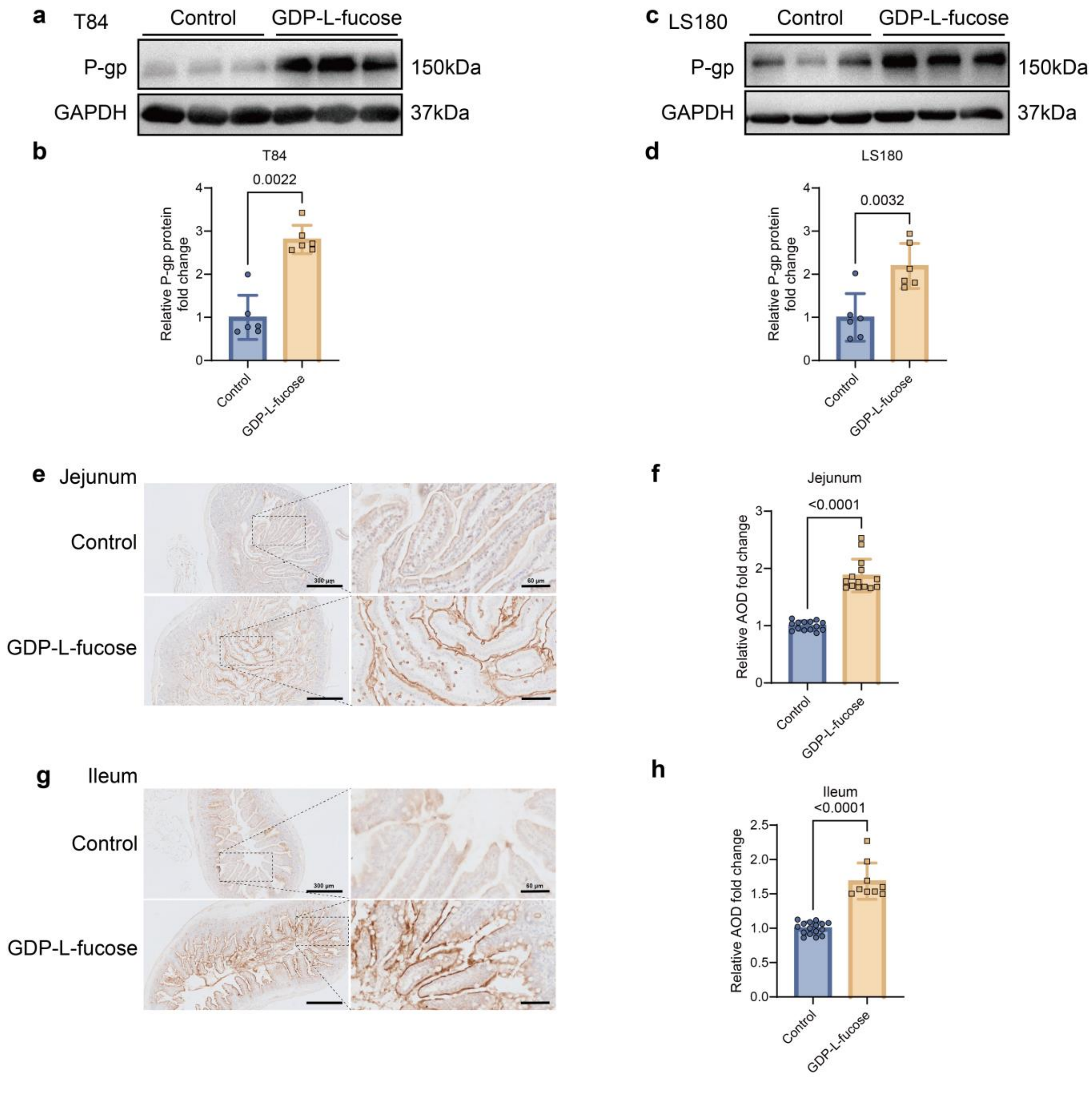

a
T84
Control
GDP-L-fucose
P-gp
150kDa
GAPDH
37kDa
b
T84
0.0022
Relative P-gp protein fold change
Control
GDP-L-fucose
c
LS180
Control
GDP-L-fucose
P-gp
150kDa
GAPDH
37kDa
d
LS180
0.0032
Relative P-gp protein fold change
Control
GDP-L-fucose
e
Jejunum
Control
GDP-L-fucose
300 μm
60 μm
f
Jejunum
<0.0001
Relative AOD fold change
Control
GDP-L-fucose
g
Ileum
Control
GDP-L-fucose
300 μm
60 μm
h
Ileum
<0.0001
Relative AOD fold change
Control
GDP-L-fucose

Figure S11

GDP-L-fucose enhances P-gp expression in T84 and LS180 cells, significantly boosts mouse intestinal P-gp expression.

a-b. Expression levels of P-gp in control T84 cells (PBS) or cells exposed to GDP-L-fucose (250μM) for 48h (n=6/group), and quantification.
c-d. Expression levels of P-gp in control LS180 cells (PBS) or cells exposed to GDP-L-fucose (250μM) for 48h (n=6/group), and quantification.
e-f. IHC staining of P-gp on jejunum sections (Control and GDP-L-fucose groups, scale bar: 300 and 60 μm), and the analysis of P-gp positive areas (n=14/group).
g-h. IHC staining of P-gp on ileum sections (Control and GDP-L-fucose groups, scale bar: 300 and 60 μm), and the analysis of P-gp positive areas (n=16 and 9/group).

Data are presented as mean ± SD. Statistical analyses were conducted using Mann-Whitney U test (b, f, h) and two-tailed unpaired t-test (d). Exact P values are reported in the figures.

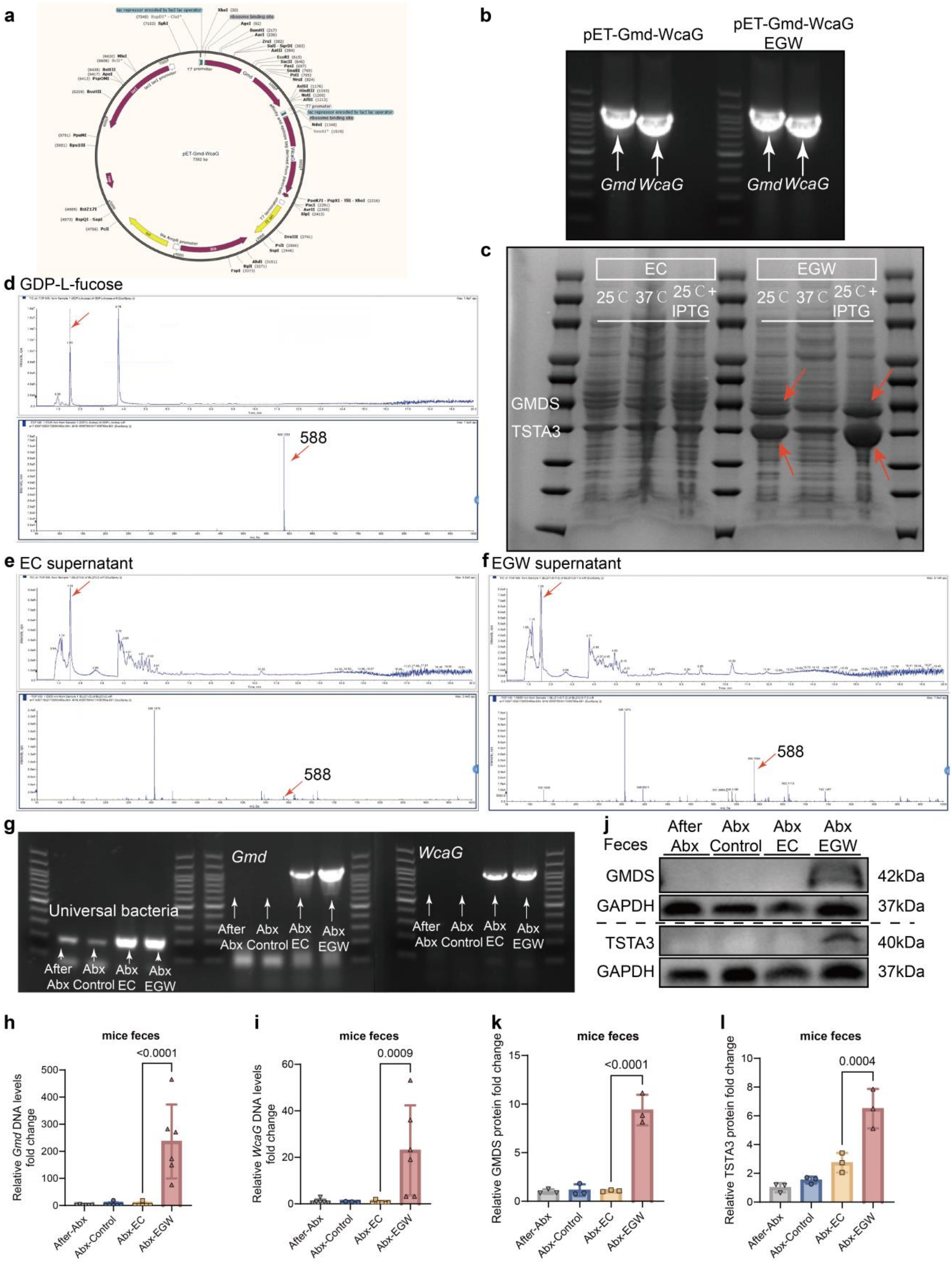
a
b
pET-Gmd-WcaG
pET-Gmd-WcaG EGW
Gmd
WcaG
Gmd
WcaG
c
EC
EGW
25 ℃
37 ℃
25 ℃+ IPTG
25 ℃
37 ℃
25 ℃+ IPTG
GMDS
TSTA3
d GDP-L-fucose
588
e EC supernatant
588
f EGW supernatant
588
g
Universal bacteria
After Abx
Abx Control
Abx EC
Abx EGW
Gmd
After Abx
Abx Control
Abx EC
Abx EGW
WcaG
After Abx
Abx Control
Abx EC
Abx EGW
j
Feces
After Abx
Abx Control
Abx EC
Abx EGW
GMDS
42kDa
GAPDH
37kDa
TSTA3
40kDa
GAPDH
37kDa
h
mice feces
<0.0001
Relative Gmd DNA levels fold change
After-Abx
Abx-Control
Abx-EC
Abx-EGW
i
mice feces
0.0009
Relative WcaG DNA levels fold change
After-Abx
Abx-Control
Abx-EC
Abx-EGW
k
mice feces
<0.0001
Relative GMDS protein fold change
After-Abx
Abx-Control
Abx-EC
Abx-EGW
l
mice feces
0.0004
Relative TSTA3 protein fold change
After-Abx
Abx-Control
Abx-EC
Abx-EGW

Figure S12

EGW yields high levels of GDP-L-fucose and effectively colonizes mice following Abx treatment.

a. Schematic diagram of the sequence of pET-Gmd-WcaG.
b. The expression of *Gmd* and *WcaG* in the DNA of pET-Gmd-WcaG and EGW by PCR.
c. The expression of GMDS and TSTA3 in the EC and EGW under different culture conditions.
d-f. Identify and detect GDP-L-fucose standard, GDP-L-fucose in EC supernatant and EGW supernatant by targeted LC/MS.
g-i. The DNA levels of *Gmd* and *WcaG* in feces of After-Abx, Abx-Control, Abx-EC and Abx-EGW groups by PCR and qPCR (n=6/group).
j-l. The protein levels of GMDS and TSTA3 in feces of After-Abx, Abx-Control, Abx-EC and Abx-EGW groups by Western blot (n=3/group).

Data are presented as mean ± SD. Statistical analyses were conducted using one-way ANOVA (h, i, k, l). Exact P values are reported in the figures.

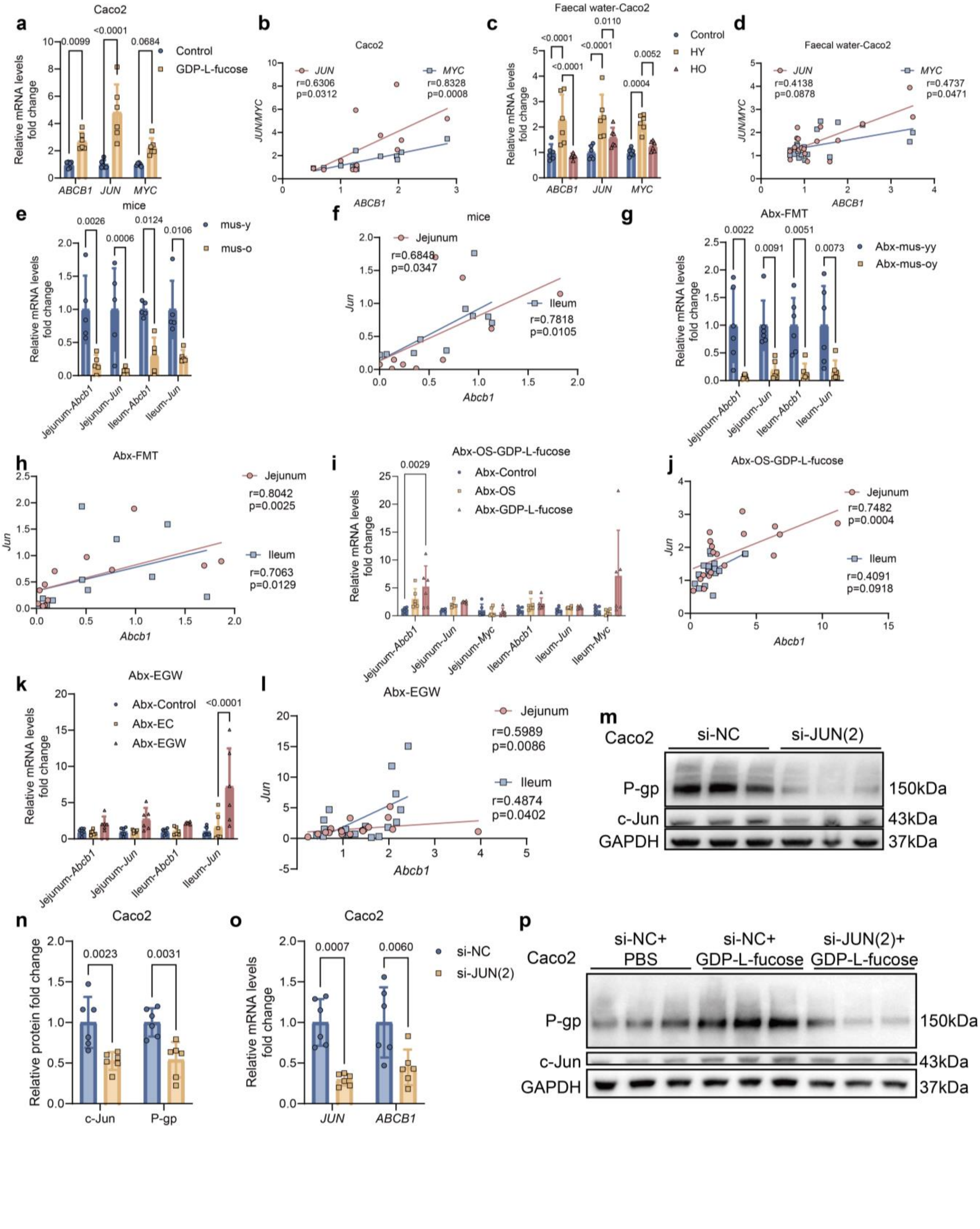
a
Caco2
Relative mRNA levels fold change
0.0099
<0.0001
0.0684
Control
GDP-L-fucose
ABCB1
JUN
MYC
b
Caco2
JUN
r=0.6306
p=0.0312
MYC
r=0.8328
p=0.0008
JUN/MYC
ABCB1
c
Faecal water-Caco2
<0.0001
0.0110
<0.0001
<0.0001
0.0052
0.0004
Control
HY
HO
Relative mRNA levels fold change
ABCB1
JUN
MYC
d
Faecal water-Caco2
JUN
r=0.4138
p=0.0878
MYC
r=0.4737
p=0.0471
JUN/MYC
ABCB1
e
mice
0.0026
0.0006
0.0124
0.0106
mus-y
mus-o
Relative mRNA levels fold change
Jejunum-Abcb1
Jejunum-Jun
Ileum-Abcb1
Ileum-Jun
f
mice
Jejunum
r=0.6848
p=0.0347
Ileum
r=0.7818
p=0.0105
Jun
Abcb1
g
Abx-FMT
0.0022
0.0091
0.0051
0.0073
Abx-mus-yy
Abx-mus-oy
Relative mRNA levels fold change
Jejunum-Abcb1
Jejunum-Jun
Ileum-Abcb1
Ileum-Jun
h
Abx-FMT
Jejunum
r=0.8042
p=0.0025
Ileum
r=0.7063
p=0.0129
Jun
Abcb1
i
Abx-OS-GDP-L-fucose
0.0029
Abx-Control
Abx-OS
Abx-GDP-L-fucose
Relative mRNA levels fold change
Jejunum-Abcb1
Jejunum-Jun
Jejunum-Myc
Ileum-Abcb1
Ileum-Jun
Ileum-Myc
j
Abx-OS-GDP-L-fucose
Jejunum
r=0.7482
p=0.0004
Ileum
r=0.4091
p=0.0918
Jun
Abcb1
k
Abx-EGW
Abx-Control
Abx-EC
Abx-EGW
<0.0001
Relative mRNA levels fold change
Jejunum-Abcb1
Jejunum-Jun
Ileum-Abcb1
Ileum-Jun
l
Abx-EGW
Jejunum
r=0.5989
p=0.0086
Ileum
r=0.4874
p=0.0402
Jun
Abcb1
m
Caco2
si-NC
si-JUN(2)
P-gp
c-Jun
GAPDH
150kDa
43kDa
37kDa
n
Caco2
0.0023
0.0031
Relative protein fold change
c-Jun
P-gp
o
Caco2
0.0007
0.0060
si-NC
si-JUN(2)
Relative mRNA levels fold change
JUN
ABCB1
p
Caco2
si-NC+ PBS
si-NC+ GDP-L-fucose
si-JUN(2)+ GDP-L-fucose
P-gp
c-Jun
GAPDH
150kDa
43kDa
37kDa

Figure S13

Significant positive correlation between intestinal *JUN* and *ABCB1*.

a. Comparison of the mRNA expression of *ABCB1*, *JUN* and *MYC* in control Caco2 cells (PBS) or cells exposed to GDP-L-fucose (250μM) for 48h (n=6/group).
b. Correlation between *JUN* and *ABCB1*, *MYC* and *ABCB1* mRNA expression levels in the Caco2 cells from **a** (n=6/group).
c. Comparison of the mRNA expression of *ABCB1*, *JUN* and *MYC* in control Caco2 cells (PBS) or cells exposed for 48 h to FW from 22-year-old adult individuals (HY-FW), and to FW from 70-year-old adult individuals (HO-FW) (n=6/group).
d. Correlation between *JUN* and *ABCB1*, *MYC* and *ABCB1* mRNA expression levels in the Caco2 cells from **c** (n=6/group).
e. Comparison of the mRNA expression of *Abcb1* and *Jun* in the jejunum and ileum of mus-y and mus-o groups (n=6/group).
f. Correlation between *Jun* and *Abcb1* mRNA expression levels in the jejunum and ileum from **e** (n=6/group).
g. Comparison of the mRNA expression of *Abcb1* and *Jun* in the jejunum and ileum of Abx-mus-yy and Abx-mus-oy groups (n=6/group).
h. Correlation between *Jun* and *Abcb1* mRNA expression levels in the jejunum and ileum from **g** (n=6/group).
i. Comparison of the mRNA expression of *Abcb1*, *Jun* and *Myc* in the jejunum and ileum of Abx-Control, Abx-OS and Abx-GDP-L-fucose groups (n=6/group).
j. Correlation between *Jun* and *Abcb1* mRNA expression levels in the jejunum and ileum from **i** (n=6/group).
k. Comparison of the mRNA expression of *Abcb1* and *Jun* in the jejunum and ileum of Abx-Control, Abx-EC and Abx-EGW groups (n=6/group).
l. Correlation between *Jun* and *Abcb1* mRNA expression levels in the jejunum and ileum from **k** (n=6/group).
m-n. Expression levels of P-gp and c-Jun in Caco2 cells transfected with si-NC or si-JUN (2) for 48 h (n=6/group), and quantification.
o. *ABCB1* and *JUN* mRNA levels of in Caco2 cells (n=6/group).
p. Expression levels of P-gp and c-Jun in Caco2 cells transfected with si-NC or si-JUN (2) and with or without GDP-L-fucose (250μM) for 48 h (n = 6/group).

Data are presented as mean ± SD. Statistical analyses were conducted using two-way ANOVA (a, c, e, g, i, k, n, o). Exact P values are reported in the figures.

Correlation analysis: Normality assessed by Shapiro-Wilk test; Pearson correlation used for normally distributed data, Spearman rank correlation for non-normal data.

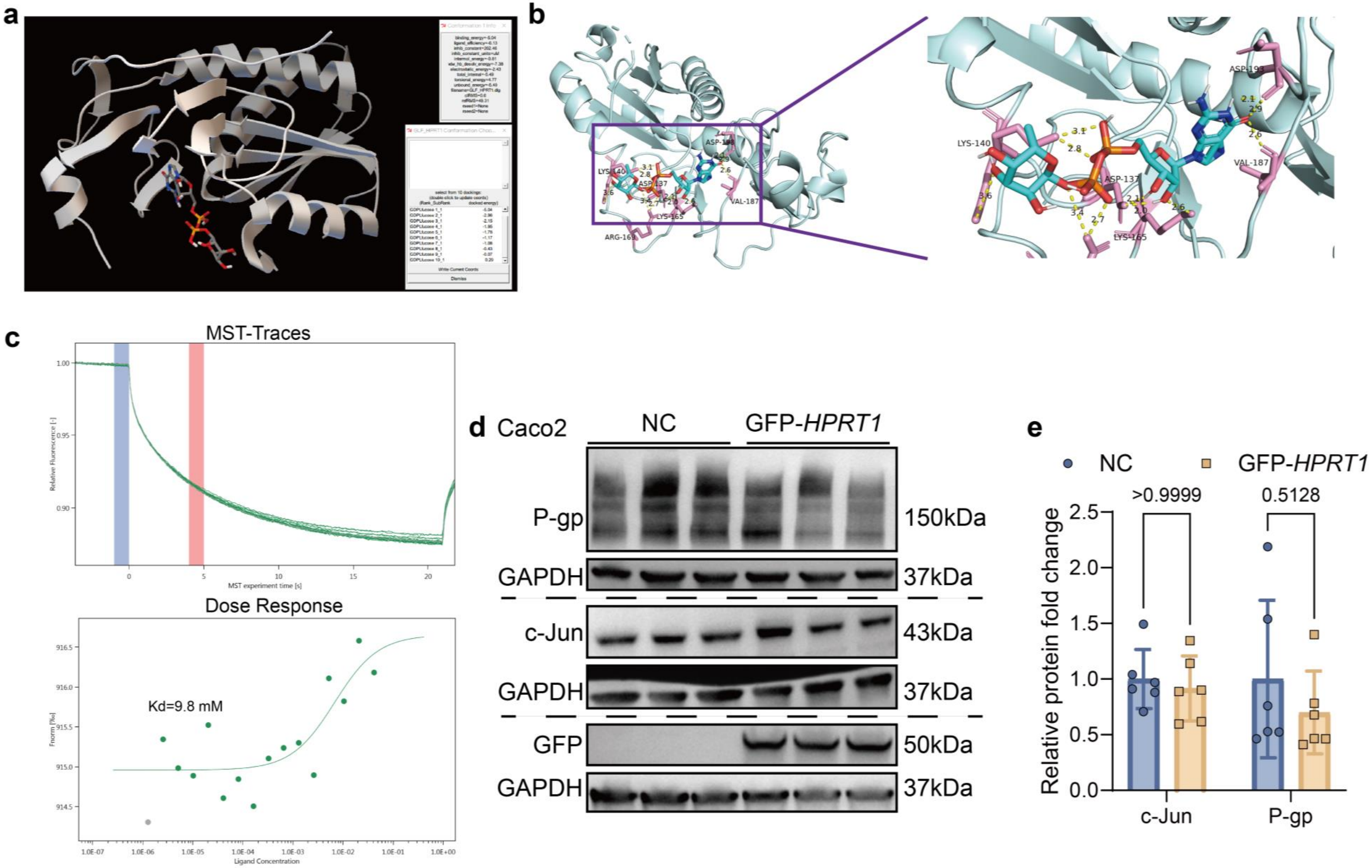
a
b
LYS-140
ASP-137
ASP-193
VAL-187
LYS-165
ARG-169
c
MST-Traces
Relative Fluorescence [-]
MST experiment time [s]
Dose Response
Kd=9.8 mM
Fnorm [‰]
Ligand Concentration
d
Caco2
NC
GFP-HPRT1
P-gp
150kDa
GAPDH
37kDa
c-Jun
43kDa
GAPDH
37kDa
GFP
50kDa
GAPDH
37kDa
e
NC
GFP-HPRT1
>0.9999
0.5128
Relative protein fold change
c-Jun
P-gp

Figure S14

GDP-L-fucose does not activate c-Jun and P-gp expression of Caco2 cells via binding with HPRT1.

a. AutoDock4.2 was utilized to achieve molecular docking between GDP-L-fucose and HPRT1.
b. Left—General view of docked GDP-L-fucose into HPRT1. Right—Zoomed-in region. The protein is a blue and pink cartoon outfit. The pink part represents the name of the specific amino acid that GDP-L-fucose bind to HPRT1, and the length and number of hydrogen bonds.
c. Binding of GDP-L-fucose to HPRT1 as analyzed by microscale thermophoresis.
d-e. Expression levels of GFP, c-Jun, and P-gp in Caco2 cells transfected with NC or GFP-*HPRT1* plasmid 48 h (n = 6/group) and quantification of c-Jun and P-gp.

Data are presented as mean ± SD. Statistical analyses were conducted using two-way ANOVA (e). Exact P values are reported in the figures.

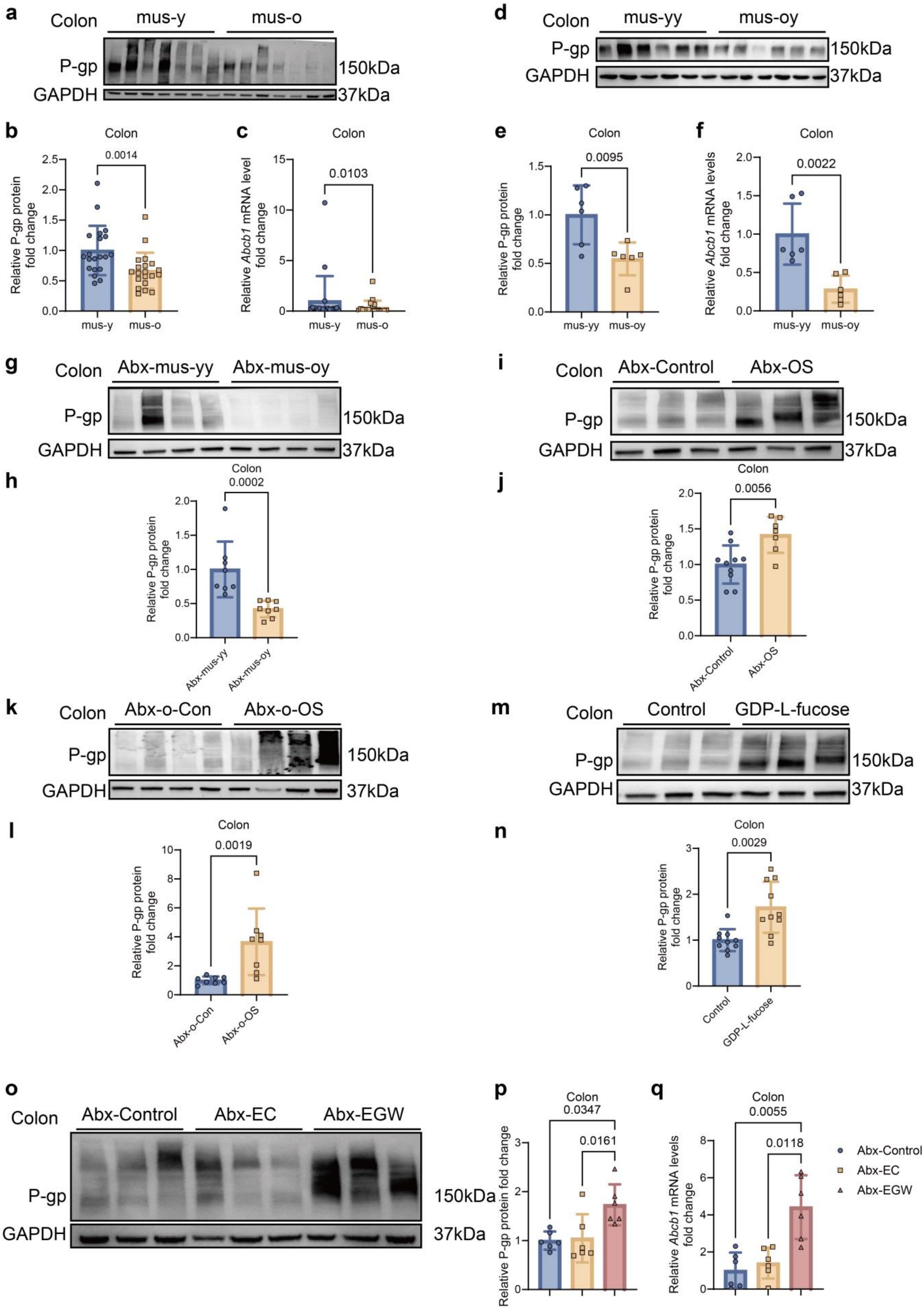

a
Colon
mus-y
mus-o
P-gp
GAPDH
150kDa
37kDa
d
Colon
mus-yy
mus-oy
P-gp
GAPDH
150kDa
37kDa
b
Colon
0.0014
Relative P-gp protein fold change
mus-y
mus-o
c
Colon
0.0103
Relative Abcb1 mRNA level fold change
mus-y
mus-o
e
Colon
0.0095
Relative P-gp protein fold change
mus-yy
mus-oy
f
Colon
0.0022
Relative Abcb1 mRNA levels fold change
mus-yy
mus-oy
g
Colon
Abx-mus-yy
Abx-mus-oy
P-gp
GAPDH
150kDa
37kDa
i
Colon
Abx-Control
Abx-OS
P-gp
GAPDH
150kDa
37kDa
h
Colon
0.0002
Relative P-gp protein fold change
Abx-mus-yy
Abx-mus-oy
j
Colon
0.0056
Relative P-gp protein fold change
Abx-Control
Abx-OS
k
Colon
Abx-o-Con
Abx-o-OS
P-gp
GAPDH
150kDa
37kDa
m
Colon
Control
GDP-L-fucose
P-gp
GAPDH
150kDa
37kDa
l
Colon
0.0019
Relative P-gp protein fold change
Abx-o-Con
Abx-o-OS
n
Colon
0.0029
Relative P-gp protein fold change
Control
GDP-L-fucose
o
Colon
Abx-Control
Abx-EC
Abx-EGW
P-gp
GAPDH
150kDa
37kDa
p
Colon
0.0347
0.0161
Relative P-gp protein fold change
q
Colon
0.0055
0.0118
Relative Abcb1 mRNA levels fold change
Abx-Control
Abx-EC
Abx-EGW

Figure S15

Young mouse-derived fecal microbiota transplantation, *O. splanchnicus*, GDP-L-fucose, or EGW promote colonic P-gp expression in mice.

a-b. Expression levels and quantitative analysis of P-gp in colon of mus-y and mus-o groups (n = 20/group).
c. *Abcb1* mRNA levels in colon of mus-y and mus-o groups were analyzed by qPCR (n = 20/group).
d-e. Expression levels and quantitative analysis of P-gp in colon of mus-yy and mus-oy groups (n = 6/group).
f. *Abcb1* mRNA levels in colon of mus-yy and mus-oy groups were analyzed by qPCR (n = 6/group).
g-h. Expression levels and quantitative analysis of P-gp in colon of Abx-mus-yy and Abx-mus-oy groups (n = 8/group).
i-j. Expression levels and quantitative analysis of P-gp in colon of Abx-Control and Abx-OS groups (n = ./group).
k-l. Expression levels and quantitative analysis of P-gp in colon of Abx-o-Con and Abx-o-OS groups (n = 8/group).
m-n. Expression levels and quantitative analysis of P-gp in colon of Control and GDP-L-fucose groups (n = 10/group).
o-p. Expression levels and quantitative analysis of P-gp in colon of Abx-Control, Abx-EC, and Abx-EGW groups (n = 6/group).
q. *Abcb1* mRNA levels in colon of Abx-Control, Abx-EC, and Abx-EGW groups were analyzed by qPCR (n = 6/group).

Data are presented as mean ± SD. Statistical analyses were conducted using Mann-Whitney U test (b, c, f, h, l, n), two-tailed unpaired t-test (e, j), and one-way ANOVA (p, q). Exact P values are reported in the figures.

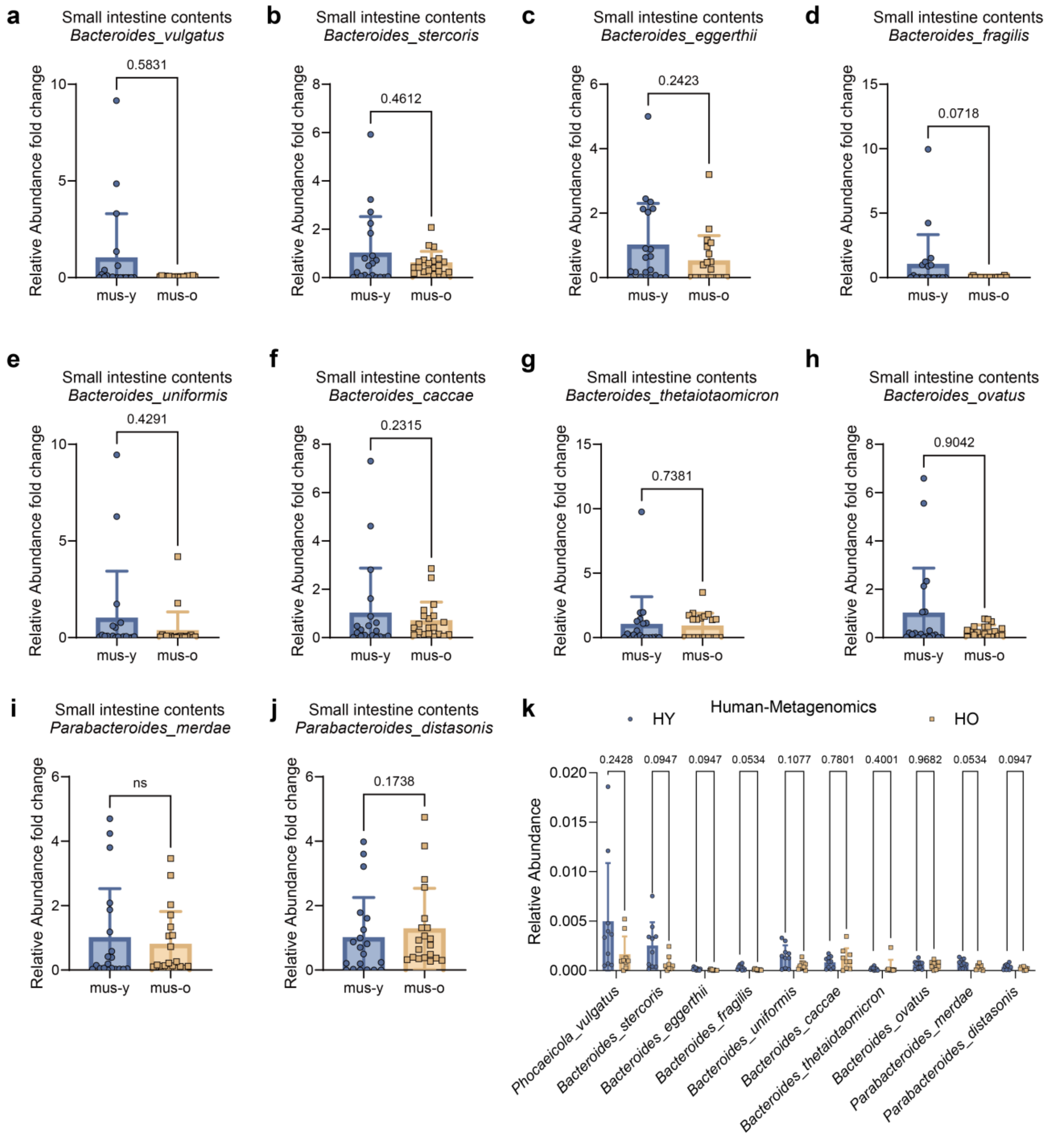
a
Small intestine contents
Bacteroides_vulgatus
0.5831
Relative Abundance fold change
mus-y
mus-o
b
Small intestine contents
Bacteroides_stercoris
0.4612
c
Small intestine contents
Bacteroides_eggerthii
0.2423
d
Small intestine contents
Bacteroides_fragilis
0.0718
e
Small intestine contents
Bacteroides_uniformis
0.4291
f
Small intestine contents
Bacteroides_caccae
0.2315
g
Small intestine contents
Bacteroides_thetaiotaomicron
0.7381
h
Small intestine contents
Bacteroides_ovatus
0.9042
i
Small intestine contents
Parabacteroides_merdae
ns
j
Small intestine contents
Parabacteroides_distasonis
0.1738
k
Human-Metagenomics
HY
HO
Relative Abundance
0.2428
0.0947
0.0947
0.0534
0.1077
0.7801
0.4001
0.9682
0.0534
0.0947
Phocaeicola_vulgatus
Bacteroides_stercoris
Bacteroides_eggerthii
Bacteroides_fragilis
Bacteroides_uniformis
Bacteroides_caccae
Bacteroides_thetaiotaomicron
Bacteroides_ovatus
Parabacteroides_merdae
Parabacteroides_distasonis

Figure S16

Other *Bacteroidetes* species exhibit no differential abundance in young vs. aged mouse small intestinal contents or human feces.

a-j. The abundance of *Bacteroides_vulgatus*, *Bacteroides_stercoris*, *Bacteroides_eggerthii*, *Bacteroides_fragilis*, *Bacteroides_uniformis*, *Bacteroides_caccae*, *Bacteroides_thetaiotaomicron*, *Bacteroides_ovatus*, *Parabacteroides_merdae*, and *Parabacteroides_distasonis* in small intestine contents bacterial DNA from mus-y and mus-o groups by qPCR (n =20/group).

k. The abundance of *Bacteroides_vulgatus*, *Bacteroides_stercoris*, *Bacteroides_eggerthii*, *Bacteroides_fragilis*, *Bacteroides_uniformis*, *Bacteroides_caccae*, *Bacteroides_thetaiotaomicron*, *Bacteroides_ovatus*, *Parabacteroides_merdae*, and *Parabacteroides_distasonis* in feces from HY and HO groups by metagenomics (n =10 and 9/group).

Data are presented as mean ± SD. Statistical analyses were conducted using Mann-Whitney U test (a-k). Exact P values are reported in the figures.

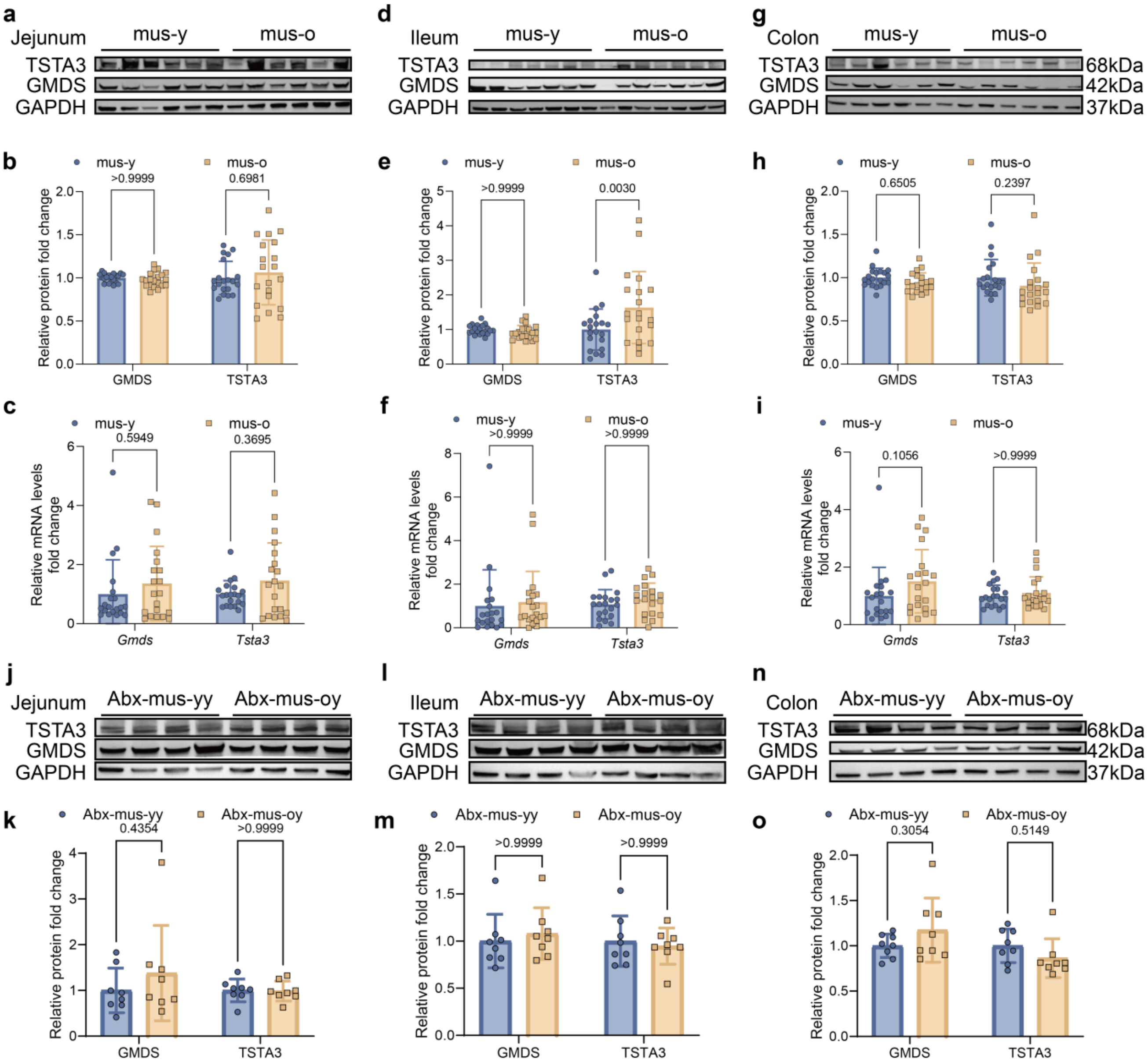

a
Jejunum
mus-y
mus-o
TSTA3
GMDS
GAPDH
d
Ileum
mus-y
mus-o
TSTA3
GMDS
GAPDH
g
Colon
mus-y
mus-o
TSTA3
GMDS
GAPDH
68kDa
42kDa
37kDa
b
mus-y
mus-o
>0.9999
0.6981
Relative protein fold change
GMDS
TSTA3
e
mus-y
mus-o
>0.9999
0.0030
Relative protein fold change
GMDS
TSTA3
h
mus-y
mus-o
0.6505
0.2397
Relative protein fold change
GMDS
TSTA3
c
mus-y
mus-o
0.5949
0.3695
Relative mRNA levels fold change
Gmds
Tsta3
f
mus-y
mus-o
>0.9999
>0.9999
Relative mRNA levels fold change
Gmds
Tsta3
i
mus-y
mus-o
0.1056
>0.9999
Relative mRNA levels fold change
Gmds
Tsta3
j
Jejunum
Abx-mus-yy
Abx-mus-oy
TSTA3
GMDS
GAPDH
l
Ileum
Abx-mus-yy
Abx-mus-oy
TSTA3
GMDS
GAPDH
n
Colon
Abx-mus-yy
Abx-mus-oy
TSTA3
GMDS
GAPDH
68kDa
42kDa
37kDa
k
Abx-mus-yy
Abx-mus-oy
0.4354
>0.9999
Relative protein fold change
GMDS
TSTA3
m
Abx-mus-yy
Abx-mus-oy
>0.9999
>0.9999
Relative protein fold change
GMDS
TSTA3
o
Abx-mus-yy
Abx-mus-oy
0.3054
0.5149
Relative protein fold change
GMDS
TSTA3

Figure S17

Intestinal GMDS and TSTA3 expression levels are unaffected by age or fecal microbiota transplantation in mice.

a-b. Expression levels and quantitative analysis of GMDS and TSTA3 in jejunum of mus-y and mus-o groups (n = 20/group).
c. *Gmd* and *Tsta3* mRNA levels in jejunum of mus-y and mus-o groups were analyzed by qPCR (n = 20/group).
d-e. Expression levels and quantitative analysis of GMDS and TSTA3 in ileum of mus-y and mus-o groups (n = 20/group).
f. *Gmd* and *Tsta3* mRNA levels in ileum of mus-y and mus-o groups were analyzed by qPCR (n = 20/group).
g-h. Expression levels and quantitative analysis of GMDS and TSTA3 in colon of mus-y and mus-o groups (n = 20/group).
i. *Gmd* and *Tsta3* mRNA levels in colon of mus-y and mus-o groups were analyzed by qPCR (n = 20/group).
j-k. Expression levels and quantitative analysis of GMDS and TSTA3 in jejunum of Abx-mus-yy and Abx-mus-oy groups (n = 8/group).
l-m. Expression levels and quantitative analysis of GMDS and TSTA3 in ileum of Abx-mus-yy and Abx-mus-oy groups (n = 8/group).
n-o. Expression levels and quantitative analysis of GMDS and TSTA3 in colon of Abx-mus-yy and Abx-mus-oy groups (n = 8/group).

Data are presented as mean ± SD. Statistical analyses were conducted using two-way ANOVA (b, c, e, f, h, i, k, m, o). Exact P values are reported in the figures.

For logical continuity and rigor, data originate from the same experiment and sample set as prior related studies.

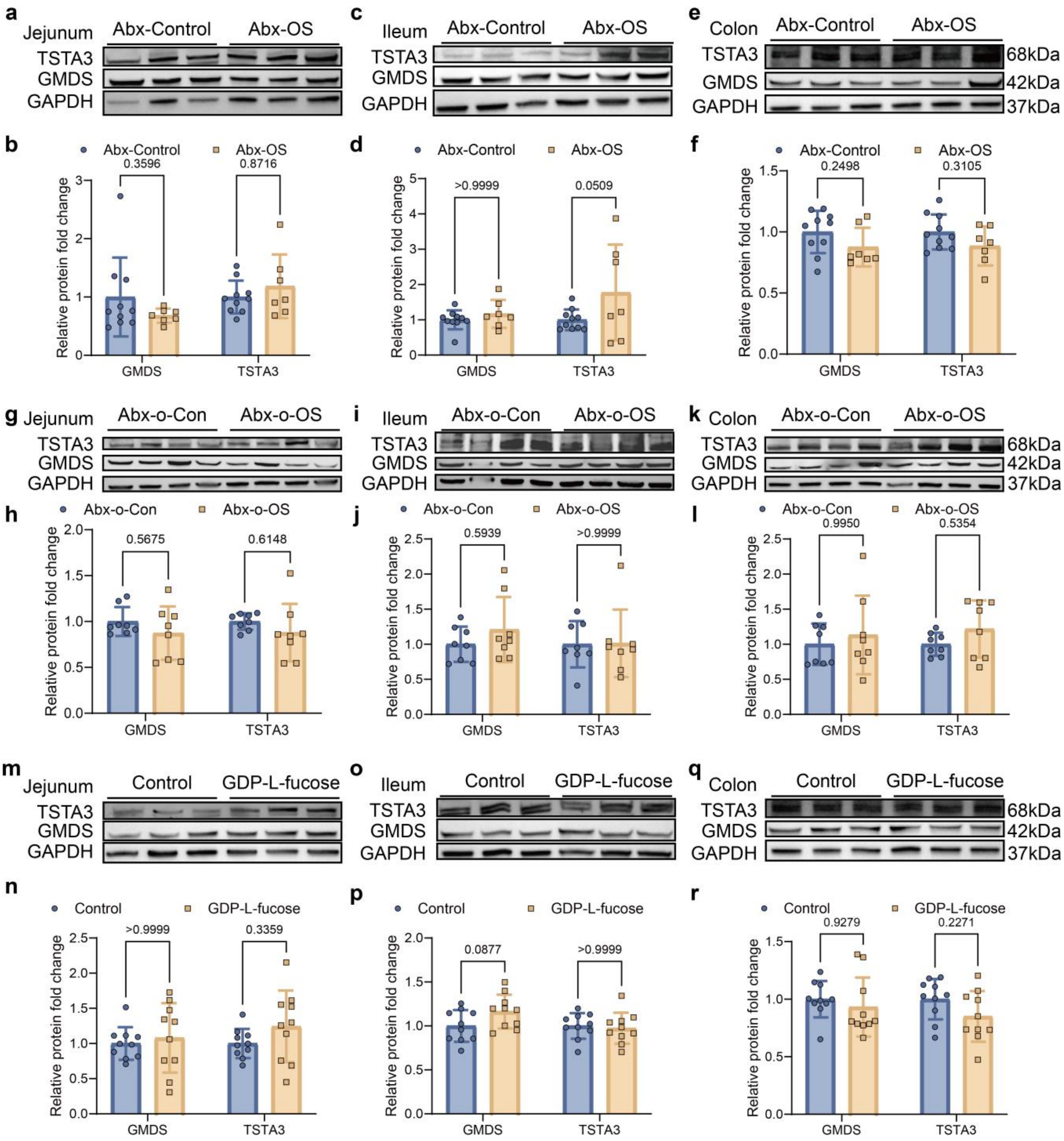

a
Jejunum
Abx-Control
Abx-OS
TSTA3
GMDS
GAPDH
b
Abx-Control
Abx-OS
0.3596
0.8716
Relative protein fold change
GMDS
TSTA3
c
Ileum
Abx-Control
Abx-OS
TSTA3
GMDS
GAPDH
d
Abx-Control
Abx-OS
>0.9999
0.0509
Relative protein fold change
GMDS
TSTA3
e
Colon
Abx-Control
Abx-OS
TSTA3
GMDS
GAPDH
68kDa
42kDa
37kDa
f
Abx-Control
Abx-OS
0.2498
0.3105
Relative protein fold change
GMDS
TSTA3
g
Jejunum
Abx-o-Con
Abx-o-OS
TSTA3
GMDS
GAPDH
h
Abx-o-Con
Abx-o-OS
0.5675
0.6148
Relative protein fold change
GMDS
TSTA3
i
Ileum
Abx-o-Con
Abx-o-OS
TSTA3
GMDS
GAPDH
j
Abx-o-Con
Abx-o-OS
0.5939
>0.9999
Relative protein fold change
GMDS
TSTA3
k
Colon
Abx-o-Con
Abx-o-OS
TSTA3
GMDS
GAPDH
68kDa
42kDa
37kDa
l
Abx-o-Con
Abx-o-OS
0.9950
0.5354
Relative protein fold change
GMDS
TSTA3
m
Jejunum
Control
GDP-L-fucose
TSTA3
GMDS
GAPDH
n
Control
GDP-L-fucose
>0.9999
0.3359
Relative protein fold change
GMDS
TSTA3
o
Ileum
Control
GDP-L-fucose
TSTA3
GMDS
GAPDH
p
Control
GDP-L-fucose
0.0877
>0.9999
Relative protein fold change
GMDS
TSTA3
q
Colon
Control
GDP-L-fucose
TSTA3
GMDS
GAPDH
68kDa
42kDa
37kDa
r
Control
GDP-L-fucose
0.9279
0.2271
Relative protein fold change
GMDS
TSTA3

Figure S18

Intestinal GMDS and TSTA3 expression levels are unaffected by *O. splanchnicus* or GDP-L-fucose in mice.

a-b. Expression levels and quantitative analysis of GMDS and TSTA3 in jejunum of Abx-Control and Abx-OS groups (n = 10 and 7/group).
c-d. Expression levels and quantitative analysis of GMDS and TSTA3 in ileum of Abx-Control and Abx-OS groups (n = 10 and 7/group).
e-f. Expression levels and quantitative analysis of GMDS and TSTA3 in colon of Abx-Control and Abx-OS groups (n = 10 and 7/group).
g-h. Expression levels and quantitative analysis of GMDS and TSTA3 in jejunum of Abx-o-Con and Abx-o-OS groups (n = 8/group).
i-j. Expression levels and quantitative analysis of GMDS and TSTA3 in ileum of Abx-o-Con and Abx-o-OS groups (n = 8/group).
k-l. Expression levels and quantitative analysis of GMDS and TSTA3 in colon of Abx-o-Con and Abx-o-OS groups (n = 8/group).
m-n. Expression levels and quantitative analysis of GMDS and TSTA3 in jejunum of Control and GDP-L-fucose groups (n = 10/group).
o-p. Expression levels and quantitative analysis of GMDS and TSTA3 in ileum of Control and GDP-L-fucose groups (n = 10/group).
q-r. Expression levels and quantitative analysis of GMDS and TSTA3 in colon of Control and GDP-L-fucose groups (n = 10/group).

Data are presented as mean ± SD. Statistical analyses were conducted using two-way ANOVA (b, d, f, h, j, l, n, p, r). Exact P values are reported in the figures.

For logical continuity and rigor, data originate from the same experiment and sample set as prior related studies.

Table S1 Summary information of subjects in metagenomic study

	Adults (18-30 years old)	Old-elderly (⩾75 years old)
No. of subjects	10	9
Demographics		
Age (years)	28.8 ± 3.19	84.3 ± 5.70
Sex, n (%)		
Male	4 (40.0)	5 (55.6)
Female	6 (60.0)	4 (44.4)
Body weight (kg)	60.2 ± 11.2	61.6 ± 12.9
eGFR (mL/min/1.73m^2)	103 ± 12.2	69.0 ± 16.5

Continuous variables are presented as mean ± SD and categorical variables are expressed as frequency counts (percentages). eGFR: estimated glomerular filtrate rate.

Table S2 Autodock prediction results of GDP-L-fucose

Protein	Minimum binding energy of conformation（KJ/mol）
Nuclear factor NF-kappa-B p105 subunit（NFKB1）	-2.33
Nuclear factor NF-kappa-B p105 subunit（NFKB1af）	0.21
Pregnane X receptor（NR1I2）	-1.81
Pregnane X receptor（NR1I2af）	-0.19
Cell division control protein 42 homolog（CDC42）	-1.37
Ras-related C3 botulinum toxin substrate 1（RAC1）	-2.35
Ubiquitin carboxyl-terminal hydrolase 7（USP7part1）	-2.95
Ubiquitin carboxyl-terminal hydrolase 7（USP7part2）	-2.32
Ubiquitin carboxyl-terminal hydrolase 7（USP7part3）	0.34
Ubiquitin carboxyl-terminal hydrolase 7（USP7af）	1.12
Adenosine A1 receptor（AA1R）	-0.67
Adenosine receptor A2a（Adora2a）	0.45
Eukaryotic translation initation factor (by homology)（eIF4E）	-5.72
DNA (cytosine-5)-methyltransferase 3B（DNMT3B）	-2.17
DNA (cytosine-5)-methyltransferase 3B（DNMT3Baf）	0.74
Telomerase reverse transcriptase（TERT）	0.14
Hypoxanthine-guanine phosphoribosyltransferase（HPRT1）	-5.04
Lymphocyte differentiation antigen CD38（CD38）	-2.07
Heat shock cognate 71 kDa protein（HSPA8）	-2.08
Heat shock cognate 71 kDa protein（HSPA8af）	-0.28
78 kDa glucose-regulated protein（HSPA5）	-2.05
78 kDa glucose-regulated protein（HSPA5af）	-0.31
MAP kinase-activated protein kinase 2（MAPKAPK2）	-2.49
Tyrosine-protein kinase SRC（SRC）	-2.64
UDP-N-acetylglucosamine--peptide N-acetylglucosaminyltransferase 110 kDa subunit（OGT）	1.23
Serine/threonine-protein kinase AKT（AKT1）	-4.66

Table S3 Primer sequences in the study

Primer name	Sequence(5'to3')
m-β-actin-F	ATCTGGCACCACACCTTC
m-β-actin-R	AGCCAGGTCCAGACGCA
m-Abcbla-F	AACGGAAGAGCAGACAAGAAC
m-Abcbla-R	CAACCTGCATAGCGAAACATTG
m-Jun-F	AAGAAGCTCACAAGTCCGGG
m-Jun-R	GAGGGCATCGTCGTAGAAGG
m-Myc-F	GCTTGGCGGGAAAAAGAAGG
m-Myc-R	CGACCGCAACATAGGATGGA
m-Muc2-F	ACCTGGAAGGCCCAATCAAG
m-Muc2-R	CTCAGCGTAGTTGGCACTCT
m-Gmds-F	TCAAGCTCCCGCTAAGTGC
m-Gmds-R	GCCAAGTACGAACCATCCTGT
m-Tsta3-F	AACCCATCGCAGGTGCAG
m-Tsta3-R	CCCATCTGCAACCACCTTCT
h-β-actin-F	GAAGATCAAGATCATTGCTCCTC
h-β-actin-R	ATCCACATCTGCTGGAAGG
h-ABCB1-F	ACTCACTTCAGGAAGCAACC
h-ABCB1-R	CGAATGAGCTCAGGCTTCCT
h-JUN-F	GTCCGAGAGCGGACCTTATG
h-JUN-R	CTTTTTCGGCACTTGGAGGC
h-MYC-F	CGTCCTCGGATTCTCTGCTC
h-MYC-R	GCTGGTGCATTTTCGGTTGT
h-ABCB1-Promoter-F	AATGTTCTGGCTTCCGTTGC
h-ABCB1-Promoter-R	GCTGATGCGCGTTTCTCTAC
Universal bacteria-F	ACTCCTACGGGAGGCAGCAGT
Universal bacteria-R	ATTACCGCGGCTGCTGGC
OS-F	ATGTAATGATGAGCACTCTAACGG
OS-R	GGCTTTTGAGATTGGCATCC
Bacteroides_vulgatus-F	CGGGCTTAAATTGCAGATGA
Bacteroides_vulgatus-R	CATGCAGCACCTTCACAGAT
Bacteroides_stercoris-F	GCTTGCTTTGATGGATGGC
Bacteroides_stercoris-R	CATGCGGGAAAACTATGCC
Bacteroides_eggerthii-F	CCCGATAGTATAGTTTTTCCGC
Bacteroides_eggerthii-R	TCCTCTCAGAACCCCTATCCAT
Bacteroides_fragilis-F	TCRGGAAGAAAGCTTGCT
Bacteroides_fragilis-R	CATCCTTTACCGGAATCCT
Bacteroides_uniformis-F	TCTTCCGCATGGTAGAACTATTA

Bacteroides_uniformis-R	ACCGTGTCTCAGTTCCAATGTG
Bacteroides_caccae-F	AAACCCATACGCCGCAAG
Bacteroides_caccae-R	GACACCTCACGGCACGAG
Bacteroides_thetaiotaomicron-F	GCAAACTGGAGATGGCGA
Bacteroides_thetaiotaomicron-R	AAGGTTTGGTGAGCCGTTA
Bacteroides_ovatus-F	TGCAAACTRAAGATGGC
Bacteroides_ovatus-R	CAAACTAATGGAACGCATC
Parabacteroides_merdae-F	AGGGTGCGTAGGTGGTGAT
Parabacteroides_merdae-R	TTCACCGCTACACCACGC
Parabacteroides_distasonis-F	TGCCTATCAGAGGGGGATAAC
Parabacteroides_distasonis-R	GCAAATATTCCCATGCGGGAT
Gmd-F	GTGTAACCGGACAAGACGGT
Gmd-R	ATACGGAGATCGCGGGTAGA
WcaG-F	CTATTGCCAAAATCGCCGGG
WcaG-R	GTACAGGTTGGTCGGCATGA

Table S4 siRNA sequences in the study

siRNA name	Sequence
JUN (1):	
Sense strand:	5'-GCUUAAACAGAAAGUCAUGTT-3'
Antisense strand	5'-CAUGACUUUCUGUUUAAGCTT-3'
JUN (2):	
Sense strand:	5'-UCAUGCUAACGCAGCAGUUTT-3'
Antisense strand:	5'-AACUGCUGCGUUAGCAUGATT-3'
The negative control siRNA sequences:	
Sense strand:	5'-UUCUCCGAACGUGUCACGUTT-3'
Antisense strand:	5'-ACGUGACACGUUCGGAGAATT-3'
EIF4E (1):	
Sense strand:	5'-CUAACCCAGAACACUAUAUTT-3'
Antisense strand:	5'-AUAUAGUGUUCUGGGUUAGTT-3'
EIF4E (2):	
Sense strand:	5'-GAGAAGCUGUUACACAUAUTT-3'
Antisense strand:	5'-AUAUGUGUAACAGCUUCUCTT-3'

Table S5 Plasmid sequences in the study

Gene name	Sequence
EIF4E	GAATTCATGGCGACTGTCGAACCGGAAACCACCCCTACTCCTAATCCCCCGA<br>CTACAGAAGAGGAGAAAACGGAATCTAATCAGGAGGTTGCTAACCCAGAAC<br>ACTATATTAAACATCCCCTACAGAACAGATGGGCACTCTGGTTTTTAAAAAT<br>GATAAAAGCAAAACTTGGCAAGCAAACCTGCGGCTGATCTCCAAGTTTGATA<br>CTGTTGAAGACTTTTGGGCTCTGTACAACCATATCCAGTTGTCTAGTAATTTA<br>ATGCCTGGCTGTGACTACTCACTTTTTAAGGATGGTATTGAGCCTATGTGGGA<br>AGATGAGAAAAACAAACGGGGAGGACGATGGCTAATTACATTGAACAAACA<br>GCAGAGACGAAGTGACCTCGATCGCTTTTGGCTAGAGACACTTCTGTGCCTT<br>ATTGGAGAATCTTTTGATGACTACAGTGATGATGTATGTGGCGCTGTTGTTAA<br>TGTTAGAGCTAAAGGTGATAAGATAGCAATATGGACTACTGAATGTGAAAAC<br>AGAGAAGCTGTTACACATATAGGGAGGGTATACAAGGAAAGGTTAGGACTTC<br>CTCCAAAGATAGTGATTGGTTATCAGTCCCACGCAGACACAGCTACTAAGAG<br>CGGCTCCACCACTAAAAATAGGTTTGTTGTTAAGGATCC
HPRT1	GAATTCATGGCGACCCGCAGCCCTGGCGTCGTGATTAGTGATGATGAACCAG<br>GTTATGACCTTGATTTATTTTGCATACCTAATCATTATGCTGAGGATTTGGAAA<br>GGGTGTTTATTCCTCATGGACTAATTATGGACAGGACTGAACGTCTTGCTCGA<br>GATGTGATGAAGGAGATGGGAGGCCATCACATTGTAGCCCTCTGTGTGCTCA<br>AGGGGGGCTATAAATTCTTTGCTGACCTGCTGGATTACATCAAAGCACTGAAT<br>AGAAATAGTGATAGATCCATTCCTATGACTGTAGATTTTATCAGACTGAAGAG<br>CTATTGTAATGACCAGTCAACAGGGGACATAAAAGTAATTGGTGGAGATGAT<br>CTCTCAACTTTAACTGGAAAGAATGTCTTGATTGTGGAAGATATAATTGACAC<br>TGGCAAAACAATGCAGACTTTGCTTTCCTTGGTCAGGCAGTATAATCCAAAG<br>ATGGTCAAGGTCGCAAGCTTGCTGGTGAAAAGGACCCCACGAAGTGTTGGA<br>TATAAGCCAGACTTTGTTGGATTTGAAATTCCAGACAAGTTTGTTGTAGGATA<br>TGCCCTTGACTATAATGAATACTTCAGGGATTTGAATCATGTTTGTGTCATTAG<br>TGAAACTGGAAAAGCAAAATACAAAGCCAAGGATCC

Supplementary references in Fig. 1A-B, Fig. S1-S4

[1] Yan, J. et al. Pharmacokinetic and pharmacodynamic drug–drug interaction assessment between pradigastat and digoxin or warfarin. The Journal of Clinical Pharma 54, 800–808 (2014).
[2] Shoaf, S. E. et al. In Vitro P-Glycoprotein Interactions and Steady-State Pharmacokinetic Interactions Between Tolvaptan and Digoxin in Healthy Subjects. The Journal of Clinical Pharmacology 51, 761–769 (2011).
[3] Vousden, M., Allen, A., Lewis, A. & Ehren, N. Lack of Pharmacokinetic Interaction between Gemifloxacin and Digoxin in Healthy Elderly Volunteers. Chemotherapy 45, 485–490 (1999).
[4] Kadokura, T. et al. Darexaban (YM150), an oral direct factor Xa inhibitor, has no effect on the pharmacokinetics of digoxin. Eur J Drug Metab Pharmacokinet 39, 1–9 (2014).
[5] Cawello, W., Mueller-Voessing, C. & Andreas, J.-O. Effect of Lacosamide on the Steady-State Pharmacokinetics of Digoxin: Results from a Phase I, Multiple-Dose, Double-Blind, Randomised, Placebo-Controlled, Crossover Trial. Clin Drug Investig 34, 327–334 (2014).
[6] Friedrich, C. et al. Evaluation of the pharmacokinetic interaction after multiple oral doses of linagliptin and digoxin in healthy volunteers. Eur J Drug Metab Pharmacokinet 36, 17–24 (2011).
[7] Awni, W. M., Hussein, Z., Cavanaugh, J. H., Granneman, G. R. & Dubé, L. M. Assessment of the Pharmacokinetic Interaction between Zileuton and Digoxin in Humans: Clinical Pharmacokinetics 29, 92–97 (1995).
[8] Stoltz, M. et al. Effect of food on the bioavailability of fexofenadine hydrochloride (MDL 16 455A). Biopharm. Drug Dispos. 18, 645–648 (1997).
[9] Mason, J., Reynolds, R. & Rao, N. The systemic safety of fexofenadine HCl. Clin Experimental Allergy 29, 163–170 (1999).
[10] Hamman, M. The effect of rifampin administration on the disposition of fexofenadine. Clinical Pharmacology & Therapeutics 69, 114–121 (2001).
[11] Bolek, T. et al. Dabigatran Levels in Elderly Patients with Atrial Fibrillation: First Post-Marketing Experiences. Drugs Aging 35, 539–544 (2018).
[12] Stangier, J., Stähle, H., Rathgen, K. & Fuhr, R. Pharmacokinetics and Pharmacodynamics of the Direct Oral Thrombin Inhibitor Dabigatran in Healthy Elderly Subjects: Clinical Pharmacokinetics 47, 47–59 (2008).
[13] Stangier, J. et al. Coadministration of Dabigatran Etexilate and Atorvastatin: Assessment of Potential Impact on Pharmacokinetics and Pharmacodynamics. Am J Cardiovasc Drugs 9, 59–68 (2009).
[14] Chaussade, E. et al. Real-Life Peak and Trough Dabigatran Plasma Measurements Over Time in Hospitalized Geriatric Patients with Atrial Fibrillation. The Journal of nutrition, health and aging 22, 165–173 (2018).
[15] Härtter, S. et al. Pharmacokinetic and pharmacodynamic effects of comedication of clopidogrel and dabigatran etexilate in healthy male volunteers. Eur J Clin Pharmacol 69, 327–339 (2013).
[16] Härtter, S., Yamamura, N., Stangier, J., Reilly, P. & Clemens, A. Pharmacokinetics and pharmacodynamics in Japanese and Caucasian subjects after oral administration of dabigatran etexilate. Thromb Haemost 107, 260–269 (2012).
[17] Testa, S. et al. Edoxaban plasma levels in patients with non-valvular atrial fibrillation: Inter

and intra-individual variability, correlation with coagulation screening test and renal function. Thrombosis Research 175, 61–67 (2019).

[18] Mendell, J., Noveck, R. J. & Shi, M. Pharmacokinetics of the Direct Factor Xa Inhibitor Edoxaban and Digoxin Administered Alone and in Combination. Journal of Cardiovascular Pharmacology Publish Ahead of Print, (2012).

[19] Diener, H.-C. et al. Antithrombotic Treatment of Embolic Stroke of Undetermined Source: RE-SPECT ESUS Elderly and Renally Impaired Subgroups. Stroke 51, 1758–1765 (2020).

[20] Lauw, M. N. et al. Effects of dabigatran according to age in atrial fibrillation. Heart 103, 1015–1023 (2017).

[21] Boehringer Ingelheim, 2010. Pradaxa (dabigatranCapsule) medicalreview(s). https://www.accessdata.fda.gov/drugsatfda_docs/nda/2010/022512Orig1s000MedR.pdfAccessed July 25, 2024

[22] Goette, A. et al. Clinical risk predictors in atrial fibrillation patients following successful coronary stenting: ENTRUST-AF PCI sub-analysis. Clin Res Cardiol 110, 831–840 (2021).

[23] Vanassche, T. et al. Impact of age, comorbidity, and polypharmacy on the efficacy and safety of edoxaban for the treatment of venous thromboembolism: An analysis of the randomized, double-blind Hokusai-VTE trial. Thrombosis Research 162, 7–14 (2018).

[24] Daiichi-Sankyo Inc., 2015, SAVAYSA(edoxaban tosylate Tablets) medical review(s). https://www.accessdata.fda.gov/drugsatfda_docs/nda/2015/206316Orig1Orig2s000MedRedt.pdf Accessed July 25, 2024

[25] Mikulík, R. et al. Frequency and Predictors of Major Bleeding in Patients With Embolic Strokes of Undetermined Source: NAVIGATE-ESUS Trial. Stroke 51, 2139–2147 (2020).

[26] Prins, M. H. et al. Oral rivaroxaban versus enoxaparin with vitamin K antagonist for the treatment of symptomatic venous thromboembolism in patients with cancer (EINSTEIN-DVT and EINSTEIN-PE): a pooled subgroup analysis of two randomised controlled trials. The Lancet Haematology 1, e37–e46 (2014).

[27] Singer, D. E. et al. Impact of Global Geographic Region on Time in Therapeutic Range on Warfarin Anticoagulant Therapy: Data From the ROCKET AF Clinical Trial. JAHA 2, e000067 (2013).

[28] Goodman, S. G. et al. Factors Associated With Major Bleeding Events. Journal of the American College of Cardiology 63, 891–900 (2014).

[29] Agnelli, G. et al. Apixaban for Extended Treatment of Venous Thromboembolism. N Engl J Med 368, 699–708 (2013).

[30] Flaker, G. C. et al. Bleeding During Treatment With Aspirin Versus Apixaban in Patients With Atrial Fibrillation Unsuitable for Warfarin: The Apixaban Versus Acetylsalicylic Acid to Prevent Stroke in Atrial Fibrillation Patients Who Have Failed or Are Unsuitable for Vitamin k Antagonist Treatment (AVERROES) Trial. Stroke 43, 3291–3297 (2012).

[31] Boehringer Ingelheim, 2010. Pradaxa (dabigatranCapsule) medicalreview(s). https://www.accessdata.fda.gov/drugsatfda_docs/nda/2010/022512Orig1s000MedR.pdf Accessed July 25, 2024
