# Supplementary material for: Odoribacter splanchnicus rescues aging-related intestinal P-glycoprotein damage via GDP-L-fucose secretion
Source: Nat Commun. 2025 Nov 27;16:10665. doi: 10.1038/s41467-025-65692-1 (PMC12660754; doi:10.1038/s41467-025-65692-1)
Supplement: Supplementary file 2 — Description of Additional Supplementary Files [file 41467_2025_65692_MOESM2_ESM.pdf]

Description of Additional Supplementary Files

File Name: Supplementary Data 1
Description: Human and mouse gut microbiota sequencing in Fig.3, Fig.S6, and Fig.S9

File Name: Supplementary Data 2
Description: Untargeted metabolomics analysis of small intestine tissue in mus-y and mus-o groups in Fig4

File Name: Supplementary Data 3
Description: RNA-seq data of Caco2 cells in Fig.6

File Name: Supplementary Data 4
Description: Database prediction in Fig.7

File Name: Supplementary Data 5
Description: Source data of all figures
