# Supplementary material for: Odoribacter splanchnicus rescues aging-related intestinal P-glycoprotein damage via GDP-L-fucose secretion
Source: Nat Commun. 2025 Nov 27;16:10665. doi: 10.1038/s41467-025-65692-1 (PMC12660754; doi:10.1038/s41467-025-65692-1)
Supplement: Supplementary file 8 — Reporting Summary [file 41467_2025_65692_MOESM8_ESM.pdf]

nature portfolio

Corresponding author(s): Dongyang Liu ; Lemin Zheng

Last updated by author(s): Sep 11, 2025

Reporting Summary

Nature Portfolio wishes to improve the reproducibility of the work that we publish. This form provides structure for consistency and transparency in reporting. For further information on Nature Portfolio policies, see our Editorial Policies and the Editorial Policy Checklist.

Statistics

For all statistical analyses, confirm that the following items are present in the figure legend, table legend, main text, or Methods section.

n/a	Confirmed	
☐	☒	The exact sample size (*n*) for each experimental group/condition, given as a discrete number and unit of measurement
☐	☒	A statement on whether measurements were taken from distinct samples or whether the same sample was measured repeatedly
☐	☒	The statistical test(s) used AND whether they are one- or two-sided<br>*Only common tests should be described solely by name; describe more complex techniques in the Methods section.*
☐	☒	A description of all covariates tested
☐	☒	A description of any assumptions or corrections, such as tests of normality and adjustment for multiple comparisons
☐	☒	A full description of the statistical parameters including central tendency (e.g. means) or other basic estimates (e.g. regression coefficient) AND variation (e.g. standard deviation) or associated estimates of uncertainty (e.g. confidence intervals)
☐	☒	For null hypothesis testing, the test statistic (e.g. *F*, *t*, *r*) with confidence intervals, effect sizes, degrees of freedom and *P* value noted<br>*Give P values as exact values whenever suitable.*
☐	☒	For Bayesian analysis, information on the choice of priors and Markov chain Monte Carlo settings
☐	☒	For hierarchical and complex designs, identification of the appropriate level for tests and full reporting of outcomes
☐	☒	Estimates of effect sizes (e.g. Cohen's *d*, Pearson's *r*), indicating how they were calculated

Our web collection on statistics for biologists contains articles on many of the points above.

Software and code

Policy information about availability of computer code

Data collection	No special software was used to collect data.
Data analysis	Software and Algorithms<br>Reveiew Manager 5.3 software RevMan, Cochrane Training, UK https://online-review-manager.com/login<br>The Simcyp Simulator v22 Certara UK Ltd., Sheffield, UK https://www.certara.com/simcyp-overview/simcyp-discovery/<br>R version 3.6.3 and 4.3.1 The R Foundation for Statistical Computing https://www.r-project.org/<br>Trimmomatic V0.36 Bolger AM, et al.,2014 http://www.usadellab.org/cms/?page=trimmomatic<br>Bowtie 2 Bolger AM, et al.,2014 https://bowtie-bio.sourceforge.net/bowtie2/index.shtml<br>OE Cloud Platform Shanghai OE Biotech Co., Ltd, Shanghai, China https://cloud.oebiotech.cn/#/home<br>MetaboAnalyst 5.0 Pang et al., 2021 https://www.metaboanalyst.ca/<br>BGI Dr.Tom BGI Genomics Co., Ltd https://biosys.bgi.com/#/report/login<br>Grundium Ocus® Grundium<br>Aperio ImageScope 12.4.6 Leica Microsystems IR GmbH<br>ImageJ Wayne Rasband<br>UCSC Genome Browser Home Fujita PA, et al., 2011 https://genome.ucsc.edu/<br>PROMO N/A http://alggen.lsi.upc.es/cgi-bin/promo_v3/promo/promoinit.cgi?dirDB=TF_8.3<br>GraphPad Prism version 8.0 N/A https://www.graphpad-prism.cn/

For manuscripts utilizing custom algorithms or software that are central to the research but not yet described in published literature, software must be made available to editors and reviewers. We strongly encourage code deposition in a community repository (e.g. GitHub). See the Nature Portfolio guidelines for submitting code & software for further information.

Data

Policy information about availability of data

All manuscripts must include a data availability statement. This statement should provide the following information, where applicable:

- Accession codes, unique identifiers, or web links for publicly available datasets
- A description of any restrictions on data availability
- For clinical datasets or third party data, please ensure that the statement adheres to our policy

All data supporting the findings of this study are publicly available and have been deposited in appropriate repositories. The microbiome, metabolomics, and RNA-seq data generated in this study have been deposited in the OMIX database at the China National Center for Bioinformation / Beijing Institute of Genomics, Chinese Academy of Sciences under accession code OMIX012289 (https://ngdc.cncb.ac.cn/omix/). These datasets, together with other supporting processed data, are also available on figshare under DOI https://doi.org/10.6084/m9.figshare.26801872.
Third-party transcriptomic data were obtained from Rasa Elmentaite et al., Wellcome Sanger Institute, as part of the publicly available resource "Cells of the human intestinal tract mapped across space and time" (https://www.gutcellatlas.org/spacetime/epithelium/), which complies with the CC BY 4.0 license terms specified by the original authors.

Research involving human participants, their data, or biological material

Policy information about studies with human participants or human data. See also policy information about sex, gender (identity/presentation), and sexual orientation and race, ethnicity and racism.

Reporting on sex and gender	We collected data for different genders, with no less than one-third of either sex. No significant gender differences were found in this study, so different genders were not analyzed separately. The gender statistics are as follows: 4 adult males, 6 adult females, 5 elderly males, and 4 elderly females.
Reporting on race, ethnicity, or other socially relevant groupings	Our study included data on all Asians and were then provided by the participants and disaggregated through their self-reports. Since this variable is the same for all subjects, we did not conduct further statistics and analysis.
Population characteristics	Our study included 10 adults with a mean age of 28.8 years and 9 older subjects with a mean age of 84.3 years.
Recruitment	Participants were informed of the clinical trial through recruitment advertisements and screened by study physicians based on protocol admission criteria. There was no self-selection bias or other bias in this study.
Ethics oversight	Peking University Third Hospital approved this clinical study.

Note that full information on the approval of the study protocol must also be provided in the manuscript.

Field-specific reporting

Please select the one below that is the best fit for your research. If you are not sure, read the appropriate sections before making your selection.

[x] Life sciences [ ] Behavioural & social sciences [ ] Ecological, evolutionary & environmental sciences

For a reference copy of the document with all sections, see nature.com/documents/nr-reporting-summary-flat.pdf

Life sciences study design

All studies must disclose on these points even when the disclosure is negative.

Sample size	Referring to multiple published life sciences studies, all biological samples ≥ 3.
Data exclusions	No data were excluded from the analyses.
Replication	All attempts at replication were successful.
Randomization	In some experiments, we used age as the grouping criterion, and except for such experiments, all allocation was random.
Blinding	The investigators were blinded to group allocation during data collection and/or analysis.

Reporting for specific materials, systems and methods

We require information from authors about some types of materials, experimental systems and methods used in many studies. Here, indicate whether each material, system or method listed is relevant to your study. If you are not sure if a list item applies to your research, read the appropriate section before selecting a response.

Materials & experimental systems

n/a	Involved in the study
☐	☒ Antibodies
☐	☒ Eukaryotic cell lines
☒	☐ Palaeontology and archaeology
☐	☒ Animals and other organisms
☐	☒ Clinical data
☒	☐ Dual use research of concern
☒	☐ Plants

Methods

n/a	Involved in the study
☒	☐ ChIP-seq
☒	☐ Flow cytometry
☒	☐ MRI-based neuroimaging

Antibodies

Antibodies used

Antibodies
c-Jun (60A8) Rabbit monoclonal antibody Cell Signaling Technology Cat#9165; RRID: AB_2130165
Histone H3 Rabbit Polyclonal Antibody Beyotime Cat#AF7101; RRID: N/A
Rabbit IgG Beyotime Cat#A7016; RRID: AB_2905533
GAPDH monoclonal antibody Proteintech Cat#60004-1-Ig; RRID: AB_2107436
anti-P Glycoprotein antibody Abcam Cat#ab170904; RRID: AB_2687930
GMDS polyclonal antibody Proteintech Cat#15442-1-AP; RRID: AB_2110816
TSTA3 polyclonal antibody Proteintech Cat#15335-1-AP; RRID: N/A
eIF4E Monoclonal antibody Cat#66655-1-Ig；RRID：AB_2882012
Rabbit monoclonal [EP2151Y] to eIF4E (phospho S209) Cat#ab76256；RRID：AB_1523534
ProteinFind® Anti-GFP Mouse Monoclonal Antibody Cat#HT801-02；RRID：AB_2922385

Validation

Paraffin Section and Immunohistochemical Staining of Intestinal Tissue
Fresh intestinal tissues were washed with PBS buffer and soaked in 4% paraformaldehyde at 4 °C for 48 hours to fix the tissues. Then, it was soaked in 20% sucrose solution for 24 hours to dehydrate the tissues. Subsequently, the intestinal tissues were embedded in paraffin, and sectioned at a thickness of 7 μm. The sections on the slides were dewaxed and rehydrated with xylene and ethanol gradients. Immunohistochemical staining of intestinal cross-sectional sections was performed using two step universal reagent kit (mouse/rabbit high-sensitivity polymer detection system) (PV-8000, ZSGB Bio) and anti-P glycoprotein antibody (ab170904, Abcam, 1:200). Immunohistochemistry images were captured by the Grundium Ocus® and processed by Aperio ImageScope 12.4.6 (Leica Microsystems IR GmbH) and ImageJ software (Wayne Rasband).
ChIP-PCR, ChIP-qPCR
Caco2 cells were cultured in a 10 cm cell culture dish with 10 mL of medium. When the cell density reached 80-90%, formaldehyde was added directly to the medium to achieve a final concentration of 1%. The mixture was then incubated at 37 °C for 10 minutes to cross-link the target protein and its corresponding genomic DNA. Enzymatic ChIP was performed using the following components: Protein A/G Magnetic Beads (P2083S, Beyotime), c-Jun (60A8) Rabbit Monoclonal Antibody (9165S, Cell Signaling Technology), Histone H3 Rabbit Polyclonal Antibody (AF7101, Beyotime) (used as a positive control), Rabbit IgG (A7016, Beyotime) (used as a negative control). ChIP-DNA samples were purified using a PCR/DNA purification kit (D0033, Beyotime). The obtained ChIP-DNA was subsequently analyzed by PCR and qPCR. Finally, the PCR products were separated by electrophoresis on a 2% agarose gel.
Western Blotting
Cellular and tissue proteins were dissociated using RIPA lysis buffer (C1053-100, APPLYGEN). This step helps to extract proteins from the cells and tissues. The following antibodies were used to label specific protein bands:
GAPDH monoclonal antibody (60004-1-Ig, Proteintech, 1:3000)
Anti-P Glycoprotein antibody (ab170904, Abcam, 1:1000)
GMDS polyclonal antibody (15442-1-AP, Proteintech, 1:500)
TSTA3 polyclonal antibody (15335-1-AP, Proteintech, 1:500)
c-Jun (60A8) rabbit mAb (9165S, Cell Signaling Technology, 1:1000)
eIF4E Monoclonal antibody（66655-1-Ig, Proteintech, 1:5000）
Rabbit monoclonal [EP2151Y] to eIF4E (phospho S209)（ab76256, Abcam, 1:2000）
ProteinFind® Anti-GFP Mouse Monoclonal Antibody (HT801-02, TransGen Biotech, 1:2000）
Horseradish peroxidase-conjugated secondary antibodies were used to bind to the primary antibodies. A chemiluminescence detection system was employed to acquire signals from the labeled protein bands.

Eukaryotic cell lines

Policy information about cell lines and Sex and Gender in Research

Cell line source(s)

Cell Lines
Human cell line: Caco2 ATCC
Human cell line:LS180 ATCC
Human cell line:T84 ATCC

Authentication

Authentication provided by the seller.

Mycoplasma contamination	All cell lines tested negative for mycoplasma contamination.
Commonly misidentified lines (See ICLAC register)	The study did not use commonly misidentified lines.

Animals and other research organisms

Policy information about studies involving animals; ARRIVE guidelines recommended for reporting animal research, and Sex and Gender in Research

Laboratory animals	Organisms/Strains<br>C57BL/6 mice Laboratory Animal Center of Peking University Health Science Center/Beijing Vital River Laboratory Animal Technology<br>Bacterial<br>Odoribacter splanchnicus BNCC BNCC359789<br>Escherichia coli DH5α N/A N/A<br>E. coli B strain BL21(DE3) Beyotime D0337<br>EGW Shanghai Newpu Biotechnology Co., Ltd N/A
Wild animals	The study did not involve wild animals.
Reporting on sex	In the preliminary stage of the study, we used a mix of male and female animals, but found that their gender had no significant impact on the experimental results. Due to the convenience of feeding, the experimental results presented in the study are all from male animals.
Field-collected samples	C57BL/6 mice were sourced from the Laboratory Animal Center of Peking University Health Science Center and Beijing Vital River Laboratory Animal Technology Co., Ltd. The mice were housed under controlled conditions at a temperature of 21 ± 1 °C and a relative humidity of 50% ± 5%, following a 12-hour light/dark cycle. They had ad libitum access to standard mouse feed and water throughout the experiments. Animal welfare was strictly ensured, and all procedures adhered to ethical regulations established by the European Parliament for the protection of animals used in scientific research.
Ethics oversight	Animal experiments adhered to regulations approved by the Institutional Animal Care and Use Committee of Peking University.

Note that full information on the approval of the study protocol must also be provided in the manuscript.

Clinical data

Policy information about clinical studies

All manuscripts should comply with the ICMJE guidelines for publication of clinical research and a completed CONSORT checklist must be included with all submissions.

Clinical trial registration	ChiCTR2100054184; NCT04743726
Study protocol	Fecal samples from young and older subjects were collected for metagenomic analysis.
Data collection	Demographic data and metagenomic assay data were collected.
Outcomes	Characteristics and differences of gut microbiota in young and older subjects.

Plants

Seed stocks	*Report on the source of all seed stocks or other plant material used. If applicable, state the seed stock centre and catalogue number. If plant specimens were collected from the field, describe the collection location, date and sampling procedures.*
Novel plant genotypes	*Describe the methods by which all novel plant genotypes were produced. This includes those generated by transgenic approaches, gene editing, chemical/radiation-based mutagenesis and hybridization. For transgenic lines, describe the transformation method, the number of independent lines analyzed and the generation upon which experiments were performed. For gene-edited lines, describe the editor used, the endogenous sequence targeted for editing, the targeting guide RNA sequence (if applicable) and how the editor was applied.*
Authentication	*Describe any authentication procedures for each seed stock used or novel genotype generated. Describe any experiments used to assess the effect of a mutation and, where applicable, how potential secondary effects (e.g. second site T-DNA insertions, mosiacism, off-target gene editing) were examined.*
